# Guidance for Canadian Breast Cancer Practice: National Consensus Recommendations for the Systemic Treatment of Patients with Triple Negative Breast Cancer in Both the Early and Metastatic Setting 2025

**DOI:** 10.3390/curroncol33050243

**Published:** 2026-04-24

**Authors:** Christine Simmons, Omar F. Khan, Christine Brezden-Masley, David W. Cescon, Anil Abraham Joy, Nathalie LeVasseur, Katarzyna J. Jerzak, Karen A. Gelmon, Sandeep Sehdev, Stephen Chia, Marc Webster, Scott Edwards, Aalok Kumar, Jeffrey Q. Cao, Jean-François Boileau, Kara Laing, Nathaniel Bouganim, Mita Manna, on behalf of Patient Advocacy, Breast Cancer Canada

**Affiliations:** 1 BC Cancer—Vancouver, University of British Columbia, Vancouver, BC V5Z 4E6, Canada; 2 Arthur J.E. Child Cancer Centre, University of Calgary, Calgary, AB T2N 5G2, Canada; 3 Marvelle Koffler Breast Centre, Mount Sinai Hospital, University of Toronto, Toronto, ON M5G 1X5, Canada; 4 Princess Margaret Cancer Centre, University of Toronto, Toronto, ON M5G 2M9, Canada; 5 Cross Cancer Institute, University of Alberta, Edmonton, AB T6G 1Z2, Canada; 6 Sunnybrook Odette Cancer Centre, University of Toronto, Toronto, ON M4N 3M5, Canada; 7 The Ottawa Hospital Cancer Centre, Ottawa, ON K1H 8L6, Canada; 8 Dr. H. Bliss Murphy Cancer Center, Memorial University of Newfoundland, St. John’s, NL A1B 3V6, Canada; 9 BC Cancer—Surrey, University of British Columbia, Vancouver, BC V5Z 4E6, Canada; 10 Jewish General Hospital, McGill University, Montreal, QC H3T 1E2, Canada; 11 McGill University Health Centre, McGill University, Montréal, QC H4A 3J1, Canada; 12 Saskatoon Cancer Centre, University of Saskatchewan, Saskatoon, SK S7N 4H4, Canada

**Keywords:** TNBC, breast cancer, Canadian consensus recommendations, evidence-informed, REAL Alliance

## Abstract

Triple-negative breast cancer (TNBC) has been associated with a poorer prognosis than other subtypes, due to its more aggressive behaviour. Since 2020, significant advances in locoregional and systemic therapy have improved outcomes for patients with TNBC, but the implementation of these treatments remains inconsistent across Canada. There is, therefore, a critical need for evidence-informed, consensus-driven guidance to support the integration of new therapies into practice. Research Excellence, Active Leadership Canadian Breast Cancer Alliance (REAL Alliance), a pan-Canadian group of breast cancer specialists and Breast Cancer Canada, a patient advocacy organization, convened to develop national clinical consensus recommendations for the management of breast cancer. Through a selective literature review and modified Delphi process of national experts in the fields of medical oncology, radiation oncology, surgical oncology and pharmacy, REAL Alliance developed national consensus recommendations for the management of TNBC. The result is a set of 23 recommendations: four overall general recommendations, 11 in early-stage TNBC, and eight in metastatic TNBC. These recommendations are intended for oncology healthcare professionals, and are intended to guide evidence-informed, consistent care across Canada.

## Simple Summary

Triple-negative breast cancer (TNBC) is a type of breast cancer that typically grows and spreads more quickly than other breast cancer subtypes. In recent years, new treatments have significantly improved outcomes for people with TNBC. As treatment options evolve, there is a need to ensure that care across Canada remains aligned with the best available evidence and delivered in a consistent manner. Research Excellence, Active Leadership Canadian Breast Cancer Alliance (REAL Alliance), in partnership with Breast Cancer Canada, developed national, expert-based recommendations to guide the management of TNBC. REAL Alliance is a pan-Canadian group of breast cancer specialists, and Breast Cancer Canada represents the patient voice. Using a review of current research and a structured consensus process involving experts in medical oncology, radiation oncology, surgical oncology, and pharmacy, the group developed 23 clinical recommendations for TNBC care. These include four general recommendations, 11 recommendations for early-stage TNBC, and eight recommendations for metastatic TNBC. These recommendations are intended to support oncology healthcare professionals in delivering evidence-based, consistent, and high-quality care for people living with TNBC across Canada.

## 1. Introduction

Breast cancer is the most prevalent cancer among women in Canada, with over 30,000 new cases reported in 2024 [1]. It accounts for roughly one quarter of all new cancer diagnoses and remains the second leading cause of cancer-related death among Canadian women [1].

Triple-negative breast cancer (TNBC) is a distinct and aggressive subtype representing approximately 10% of all breast cancer cases in Canada, and 20% of breast cancer cases in women under the age of 40 [2]. TNBC is associated with a higher risk of early relapse, a greater incidence of germline breast cancer gene (*BRCA*) mutations, and poorer outcomes compared to other subtypes [2]. Historically, chemotherapy has been the mainstay of treatment for TNBC, and for many years, the TNBC treatment landscape remained stagnant. However, recent advances—including immunotherapies, antibody drug conjugate (ADC) therapies, and poly-adenosine ribonucleotide polymerase (PARP) inhibitors—have expanded the range of therapeutic possibilities. While this progress is encouraging, the pace and number of advancements has made clinical decision-making increasingly complex. Ensuring that high-quality care is received by all patients requires careful consideration of the evolving evidence and patient-specific factors.

There is a clear need for structured, evidence-informed guidance to support oncology teams in navigating these options and delivering optimal care. As noted in our previous publications [3,4] and restated here, Research Excellence, Active Leadership Canadian Breast Cancer Alliance (REAL Alliance) is a standing nucleus committee of multi-disciplinary, clinical–academic oncologists and other specialists in breast cancer from across Canada, in addition to representatives from Breast Cancer Canada, a patient advocacy organization. Formed in December 2023, REAL Alliance provides timely, evidence-informed recommendations for breast cancer treatment, ensuring consistent and informed care across Canada. Building on prior publications in this series, the present manuscript provides guidance for the systemic treatment of TNBC, offering a framework that integrates tumour biology, clinical stage, and patient preferences into shared decision-making.

## 2. Materials and Methods

### Consensus Recommendation Process

The methods are previously published [3,4] and are restated here. A targeted, non-systematic literature search was conducted to identify evidence relevant to the treatment of TNBC. Searches were performed in PubMed (which includes MEDLINE-indexed content), using selected terms related to TNBC and systemic therapies, including chemotherapy, immunotherapy, antibody–drug conjugates, and treatment setting (neoadjuvant, adjuvant, metastatic). The review focused on randomized trials, meta-analyses, and large observational studies published since 2010. Abstracts from major oncology meetings in 2024 and 2025 (American Society of Clinical Oncology [ASCO], European Society for Medical Oncology [ESMO], San Antonio Breast Cancer Symposium [SABCS]) and international clinical practice guidelines were also reviewed to capture emerging data. The search strategy is presented in Table S1. The evidence was synthesized qualitatively, with expert clinical interpretation applied in areas where high-level evidence was limited.

Three members of REAL Alliance (C.S., O.F.K, C.B.-M.) lead this work as a sub-committee, providing an overview and highlighting key clinical areas of need for consensus statements and the relevant evidence related to these clinical areas. The sub-committee developed the preliminary recommendations, and the most relevant references from the literature review were selected and linked to each recommendation. The statements were then revised based on feedback and discussion at a two-day, in-person consensus meeting in Toronto, ON, Canada, in April 2025. These recommendations were then subjected to a modified Delphi process in which an initial round of anonymous voting was carried out by an expert panel using an electronic platform. This panel consisted of 13 medical oncologists, one surgical oncologist, one radiation oncologist, one oncology pharmacist, and one patient advocacy representative from Breast Cancer Canada, all with specialized expertise and experience in managing breast cancer. During the voting process, panellists anonymously indicated their level of agreement with each statement, provided suggestions for revisions, and commented on specific references (including any missing references from the literature review) and background data. Further targeted literature searches were conducted to further inform and support the recommendations.

The revised statements were then subjected to a second round of anonymous electronic voting where participants evaluated their agreement with each statement after reviewing a summary of the discussion and the corresponding references. Based on our prior methodology, a recommendation is considered to be accepted if 75% or more of the participants indicated agreement after two to three rounds of voting. The recommendations in this document achieved this outcome after two rounds of voting. The voting summary appears in Table S2.

The strength of each of the recommendations followed the same criteria as utilized in REAL Alliance in previous publications [3,4] and are defined as follows:

- **A strong recommendation** is based on the highest quality of evidence.
- **A moderate recommendation** is supported by evidence that is less robust, indirect, or limited in scope.
- **A weak recommendation** is based on lower quality evidence.
- **Expert opinion** reflects recommendations by REAL Alliance for which there is limited evidence.

As a final step, the sub-committee compared the current ESMO and ASCO guidelines to REAL consensus recommendations and recorded consistencies or inconsistencies in tabular form.

## 3. Results

### General Recommendations

The following general recommendations (Table 1) outline foundational principles that apply across the TNBC care continuum, including tumour classification, genetic risk assessment, treatment timing, and shared decision-making. These principles provide the framework upon which stage- and treatment-specific recommendations are subsequently built.

**Recommendation 1:** 
*For all aspects of care for patients with TNBC, shared decision-making in partnership with patients and their caregivers is essential. All subsequent recommendations should be applied within the context of the patient’s preferences, values, and goals, ensuring a collaborative approach to treatment planning [Expert opinion].*

All recommendations in this document should be applied in the context of each patient’s individual circumstances. Clinicians play a critical role in providing clear, accurate, and balanced information to support informed decision-making that reflects patients’ values, goals of care, and lived realities. Attention to language, cultural, financial, and geographic barriers is essential to promote equitable access to high-quality cancer care. Creating a safe and supportive environment that empowers patients to articulate their priorities is fundamental for shared decision-making and true partnership in care.

Clinical trial participation should be actively considered and discussed at every appropriate decision point along the TNBC care pathway. Trials offer patients access to emerging therapies and may provide enhanced clinical oversight, structured follow-up, and additional supportive resources that are inherent to research participation. While these elements do not uniformly translate into improved clinical outcomes, they may contribute to patient experience and care delivery [10]. Overcoming barriers to trial access and accrual, and ensuring the equitable availability of research opportunities, should be viewed as a core responsibility in the care of patients with TNBC.

**Recommendation 2:** 
*Breast cancers with low hormone receptor expression, specifically ER ≤ 10%, PR ≤ 10%, and lack of HER2 overexpression or gene amplification (IHC ≤ 1 + or 2 + with negative ISH) should preferentially be treated as TNBC [Moderate recommendation].*

Multiple large, retrospective, registry-based, and real-world studies (summarized in Table 2) demonstrate that tumours with estrogen receptor (ER)-low (1–10%), progesterone receptor (PR)-low, and human epidermal growth factor receptor 2 (HER2)-negative status share clinicopathologic features and chemosensitivity that closely resemble classical TNBC when treated with chemotherapy-based regimens. As well, outcomes for ER-low tumours consistently show pathologic complete response (pCR) rates and survival outcomes that are similar to ER-negative (0%) disease and are clearly distinct from cancers that are ER > 10%, supporting the alignment of systemic treatment strategies with TNBC principles. Although ER-low tumours are often managed according to TNBC treatment principles, it is important to recognize that most prospective immunotherapy trials in TNBC primarily enrolled patients with ER-negative disease (e.g., <1%). As a result, the benefit of immune checkpoint inhibition in ER-low tumours is largely extrapolated from classical TNBC populations, and prospective validation in this subgroup is limited.

ER-low disease represents a biologically heterogeneous group, while pooled analyses show limited overall benefit from endocrine therapy (ET) in ER 1–10% tumours, more recent registry studies suggest a modest survival benefit in selected patients, particularly those with ER expression in the 6–10% range or with residual disease after neoadjuvant chemotherapy (NAC) [11]. The role of adjuvant ET in such ER-low cases is unclear. While ET may be offered after chemotherapy, particularly in patients with ER expression closer to 10% or those with residual disease after NAC, the expected benefit is likely modest. In patients whose tumours behave clinically and biologically like TNBC, the omission of ET may be reasonable, with careful counselling regarding the uncertain and possibly small magnitude of benefit weighed against potential toxicities.

**Table 2:** Summary of evidence in ER-Low (1–10%), HER2-negative breast cancer.

Study (Year)	Data Source/Design	Population (ER Definition)	Treatment Context	Key Findings	Relevance to Recommendation
Acs et al., 2024 [12]	Swedish population-based real-world cohort	HER2-negative tumours treated as TNBC with ER < 10%; ER-zero *n* = 5095 vs. ER-low (1–9%) *n* = 560	Standard TNBC management	pCR rate ER < 10% 28.1% vs. ER-zero 25.1%; no difference in OS after adjustment (HR 1.11, 95% CI 0.90–1.36)	**Supports that ER-low (1–9%) tumours behave like TNBC** when treated as TNBC
Bonadio et al., 2025 [13]	Neo-Real/GBECAM-0123 real-world cohort	ER-low (1–10%), HER2-negative, Stage II–III (*n* = 20)	Neoadjuvant pembrolizumab + chemotherapy	pCR rate 60%; Fewer pembrolizumab cycles associated with lower pCR	Supports treating ER-low/HER2-negative disease according to **TNBC neoadjuvant immunotherapy standards**
Cardoso et al., 2025 [14]	KEYNOTE-756	ER positivity: ER ≥ 10% *n* = 1201 (94%) ER < 10% *n* = 77 (6%)	Neoadjuvant pembrolizumab 200 mg or placebo Q3W in combination with standard chemotherapy regimens	Overall: pCR 24.3% vs. 15.6% (+8.5 pp, 95% CI 4.2–12.8); ER < 10% subgroup: pCR 55.9% vs. 30.2% (+25.6 pp, 95% CI 3.3–45.8); ER ≥10% subgroup: pCR 22.5% vs. 14.5% (+8.0 pp, 95% CI 3.6–12.4)	**pCR was numerically higher** in subgroup of patients with ER < 10%
Moldoveanu et al., 2023 [15]	NCDB real-world cohort	HER2-negative BC; ER-negative (13.8%) vs. ER ≤ 10% (2.0%) vs. ER > 10% (84.2%) (*n* ≈ 233,000)	Real-world chemotherapy and NAC use	pCR with NAC in ER ≤ 10% ~38–40%, similar to ER-negative and far higher than ER > 10% ~8%	Strong evidence that **ER-low tumours are biologically and clinically closer to TNBC**
Chen et al., 2018 [16]	Systematic review and meta-analysis (6 studies)	ER 1–9% vs. ER-negative vs. ER ≥10% (*n* = 16,606)	ET vs. no ET	ER 1–9% gained no significant survival benefit from ET; endocrine responsiveness significantly lower than ER ≥ 10%	Supports **limited endocrine sensitivity** and cautions against managing ER-low as conventional HR+ disease
Choong et al., 2025 [11]	NCDB real-world	Stage I–III ER-low (1–10%), received chemotherapy (*n* = 7018)	Adjuvant ET vs. ET omission	ET omission associated with worse OS (HR 1.23); effect strongest in ER 6–10% and in patients with residual disease after NAC	Demonstrates **modest but clinically relevant endocrine benefit** in selected ER-low patients, supporting offering ET with counselling
Xie et al., 2022 [17]	Prospective cohort	ER-low (1–10%) early breast cancer (*n* = 407)	Adjuvant ET (AI, TAM)	ET improved BCSS in ER-low: 5-y BCSS 94.7–93.2% with ET vs. 87.9% without; HR 0.41 (95% CI 0.19–0.91, *p* = 0.02), strongest with AI-based therapy (HR 0.36, *p* = 0.04)	Reinforces that **ER-low tumours retain endocrine sensitivity**, though the magnitude of benefit is smaller than classic ER+

Abbreviations: AI = aromatase inhibitor; BC = breast cancer; ER = estrogen receptor; ET = endocrine therapy; HER2 = human epidermal growth factor receptor 2; HR = hazard ratio; HR+ = hormone receptor-positive; NAC = neoadjuvant chemotherapy; NCDB = National Cancer Database; OS = overall survival; pCR = pathologic complete response; TAM = tamoxifen; and TNBC = triple-negative breast cancer.

International guidelines reflect this nuance. The ASCO/College of American Pathologists (CAP) guidelines define 1–10% ER staining as “ER-low positive” and recommends that pathologists report the uncertain but possible benefit of ET [8]. Similarly, ESMO, the National Comprehensive Cancer Network (NCCN), and recent expert reviews recognize ER-low tumours as being biologically closer to TNBC, supporting that chemotherapy decisions (and, where appropriate, immunotherapy) follow TNBC principles, but generally advise that adjuvant ET should not be withheld when toxicity is acceptable, especially in node-positive or higher-risk disease [5,18]. In contrast, data for adjuvant cyclin-dependent kinase 4 and 6 (CDK4/6) inhibitors in ER-low disease are extremely limited, as these patients were underrepresented or excluded from pivotal trials. Furthermore, molecular and clinicopathologic studies suggest that ER-low tumours exhibit basal-like or non-luminal biology with limited endocrine dependence [19], which further supports not using CDK4/6 inhibitors in this subtype. Given the uncertain benefit and additional toxicity, we do not recommend that CDK4/6 inhibitors be added in the adjuvant setting, based solely on the ER-low status [20,21].

Assessment of ER and PR expression is subject to technical and interpretive variability, particularly in tumours with low-level receptor staining near clinically meaningful cutoffs [22]. Immunohistochemical (IHC) results are influenced by pre-analytic handling, analytic methods, and subjective estimation of the staining percentage [8]. A multidisciplinary review is therefore encouraged in cases of uncertainty to ensure accurate classification of ER-low and PR-low disease and to guide appropriate systemic treatment decisions.

**Recommendation 3:** 
*Germline hereditary cancer multi-gene panel testing should be offered to all patients with a diagnosis of TNBC, regardless of age, stage, or family history [Strong recommendation].*

Compared with other breast cancer subtypes, TNBC carries a higher prevalence of pathologic germline variants. While only ~5% of all breast cancers are associated with inherited predisposition mutations, large-scale studies show that 15–16% of patients with TNBC carry such variants [23,24]. Historically, testing prioritized *BRCA1/2* variants. However, other predisposition genes (including *PALB2*, *RAD51C/D*, and *BARD1)* also occur at meaningful frequencies in TNBC and may have clinical implications for treatment, surveillance, surgical decisions, and family risk management [25]. International guidance emphasizes the early use of multigene panels for assessing germline mutation status, beyond simply *BRCA1/2*, to ensure that results are complete and available to help with surgical and adjuvant treatment decision-making [9,26,27,28,29].

REAL Alliance voted in support of universal hereditary cancer gene panel testing for TNBC. In Canada, an expert working group has proposed a practical framework designed for a decentralized and capacity-constrained healthcare environment for all subtypes of breast cancer [30]. Their recommendations include using standardized multigene panels, testing regardless of age or family history, using mainstreaming pathways, aligning turnaround times with the timing of surgical and adjuvant treatment decisions, and implementing genetic counselling safeguards. Although REAL Alliance did not vote to formally endorse this specific model, we acknowledge that system changes will be necessary to ensure that our recommendation can be delivered efficiently and equitably across Canada.

**Recommendation 4:** 
*Patients with early-stage TNBC should receive initial treatment (surgery or systemic neoadjuvant therapy) as quickly as possible, ideally within 30 days of biopsy result, recognizing that timely treatment initiation is particularly important, given the aggressive biology of TNBC [Strong recommendation].*

Across large U.S. population-based cohorts (Surveillance, Epidemiology, and End Results [SEER]–Medicare, *n* = 94,544; National Cancer Database [NCDB], *n* = 115,790), longer time to surgery in patients with breast cancer was consistently associated with worse survival in Stage I–II disease [31]. In a 2025 meta-analysis, it was found that each stepwise 30-day increase in time to surgery was associated with a 9–10% increase in the hazard of death, with a statistically significant association for Stage I and II disease [32]. While these reports were not specific to TNBC, other NCDB data indicate that the effect of delay does not differ by subtype [33]. A Swedish study (7017 patients, 2001–2008) found that surgery at 6 weeks after diagnosis conferred a 1.26-fold increased hazard rate of death compared to surgery at 3 weeks after diagnosis [34]. The association between overall survival (OS) and time-to-surgery was assessed in a universal-access U.S. Military Health System database (*n* = 9669) [35]. There was a higher risk of mortality associated with time-to-surgery ≥36 days (hazard ratio [HR] 1.30; 95% CI 1.04, 1.61) [35]. Another recent meta-analysis demonstrated a 10% increase in the risk of breast-cancer related death for every four week delay in treatment initiation [32]. Taken together, these data support timely therapy, ideally within 30 days of diagnosis, to minimize the adverse effects on survival that are associated with incremental treatment delays.

With regard to the timing of NAC in TNBC, a similar association was seen in an analysis using the Tumor Centre Regensburg clinical cancer registry database, which demonstrated that patients who received NAC ≤14 days after diagnosis had an estimated mean OS of 8.4 years, while patients who received NAC after more than 56 days had an estimated mean OS of 3.3 years [36].

Consistent with these studies, ESMO guidelines stress prompt therapy initiation, though without specifying the time to treatment [37]. Further to this, the World Health Organization (WHO) identified that disparities in breast cancer outcomes in low- and middle-income countries are largely based on factors that affect timely access to care, resulting in late diagnosis and delays in treatment initiation. As such, they have developed the Global Breast Cancer Initiative, with targets of 30 days from symptom presentation to diagnosis, and overall 60 days from abnormal imaging results to initiation of treatment (surgery or systemic therapy) [38].

In Canada, several provinces provide guidance on time-to-surgery for aggressive malignancies, but these recommendations are based on a metric of *time from ready-to-treat to treatment*, which is largely criticized by clinicians as lacking clinical relevance, functioning more as a healthcare system metric rather than a quality of cancer care metric [39]. Several provincial cancer organizations are now adopting the more clinically relevant quality metrics of *time from symptoms or abnormality to diagnosis*, and *time from diagnosis to first treatment (surgical or systemic)*.

Accordingly, REAL Alliance endorses prompt initiation of the first treatment (surgery or systemic neoadjuvant therapy), ideally within 30 days of the diagnosis. Where feasible, parallel care pathways, including synchronous staging, fertility counselling, preparation for systemic therapy, and initiation of germline genetic testing, should be implemented to avoid unnecessary delays. As per the WHO Global Breast Cancer Initiative, a total of 60 days from diagnosis is considered acceptable in low- and middle-income countries, but continuing to strive for decreased delays will improve not only patient survival but the overall outcomes and costs to the system in the long term.

## 4. Early TNBC Systemic Therapy

Systemic therapy for early (Stage I–III) TNBC takes into consideration the need to treat aggressively when risk is high and consider the modification of strategies where benefit may be marginal. For the purposes of these consensus recommendations, the treatment recommendations for early-stage TNBC (Table 3) are divided as follows:

- **Stage I disease** (T1a–T1c, node-negative), where the focus is on selecting patients for adjuvant or, in some cases, NAC, and avoiding overtreatment in those with very small, low-risk tumours.
- **Stage II–III disease**, where systemic therapy is typically initiated in the neoadjuvant setting and adjuvant escalation strategies are guided by the residual disease burden following surgery.
- **Follow-up, surveillance and special populations** with ER-low and uncommon histological subtypes.

**Figure 1:**
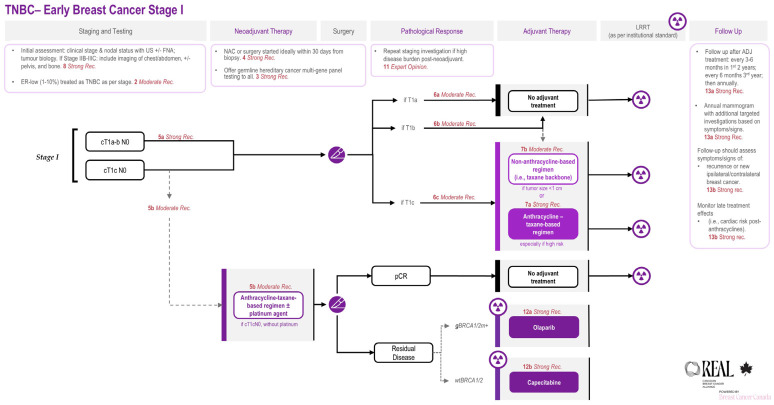
**REAL Alliance recommendations for the management of Stage I early TNBC.** Abbreviations: AC = doxorubicin–cyclophosphamide; ADJ = adjuvant; AJCC = American Joint Committee on Cancer; Cb = carboplatin; cN = clinical nodal stage (per AJCC Cancer Staging Manual, 8th edition); cT = clinical tumour stage (per AJCC Cancer Staging Manual, 8th edition); ER = estrogen receptor; gBRCA1/2m+ = germline BRCA1/2 pathogenic variant positive; LRRT = locoregional radiotherapy; NAC = neoadjuvant chemotherapy; N+ = node-positive; pCR = pathologic complete response; Rec. = recommendation; and TNBC = triple-negative breast cancer; wtBRCA1/2 = BRCA1/2 wild-type. Residual Disease = non-pCR, pathological evidence of invasive disease persists in surgical specimen of breast and/or lymph nodes.

**Figure 2:**
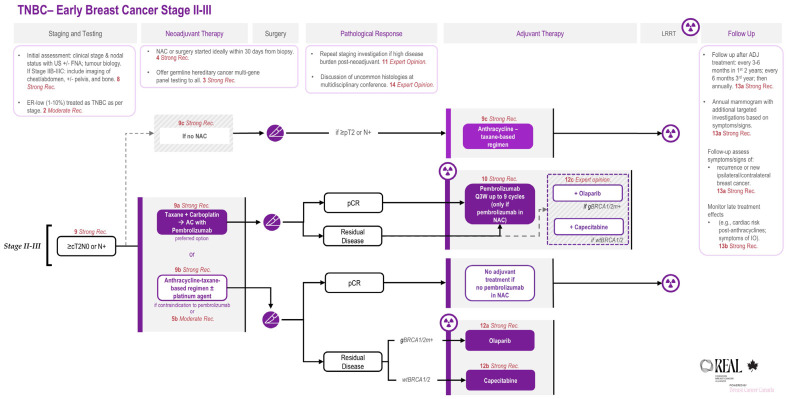
**REAL Alliance recommendations for the management of Stage II–III early TNBC.** Abbreviations: AC = doxorubicin–cyclophosphamide; ADJ = adjuvant; AJCC = American Joint Committee on Cancer; cN = clinical nodal stage (per AJCC Cancer Staging Manual, 8th edition); p/cT = pathological/clinical tumour stage (per AJCC Cancer Staging Manual, 8th edition); ER = estrogen receptor; FNA = fine needle aspiration; gBRCA1/2m+ = germline BRCA1/2 pathogenic variant positive; IO = immuno-oncology; LRRT = locoregional radiotherapy; NAC = neoadjuvant chemotherapy; N+ = node-positive; pCR = pathologic complete response; Q3W = every 3 weeks; Rec. = recommendation; TNBC = triple-negative breast cancer; US = ultrasound; and wtBRCA1/2 = BRCA1/2 wild-type.

The corresponding treatment algorithms for early-stage TNBC are illustrated in Figure 1 and Figure 2.

**Table 3:** Summary of REAL Alliance recommendations for treatment of TNBC early breast cancer.

Recommendations for Early Systemic Treatment of TNBC		REAL	ESMO [5]	ASCO [40,41,42]
*Stage I*				
**5**	**For patients with Stage I TNBC whose disease is node-negative, confirmed by ultrasound or ultrasound-guided FNA prior to surgery, and for situations where the clinical stage of the tumour is as follows:** a) ≤1 cm (T1a, b), upfront surgery is the standard of care. b) >1.0 cm and ≤2 cm (T1c), NAC (without immunotherapy) could be considered.	Strong recommendation ●●● Moderate recommendation ●●	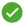 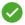	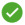 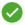
**6**	**For patients with Stage I TNBC who undergo upfront surgery and whose tumour is as follows:** a) ≤0.5 cm (T1a), adjuvant chemotherapy is not recommended. b) >0.5 cm and ≤1.0 cm (T1b), adjuvant chemotherapy could be considered through shared decision-making. c) >1.0 cm and ≤2 cm (T1c), adjuvant chemotherapy (without immunotherapy) should be considered.	Moderate recommendation ●● Moderate recommendation ●● Moderate recommendation ●●	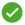 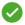 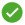	NC NC NC
**7**	**For patients with Stage I (T1b–T1c, N0) TNBC receiving chemotherapy:** a) Standard therapy should include a taxane (e.g., paclitaxel or docetaxel). b) To reduce the risk of long-term cardiotoxicity, anthracycline-containing regimens could be avoided for tumours ≤ 1 cm.	Strong recommendation ●●● Moderate recommendation ●●	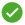 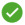	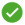 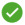
** *Stage II–III* **				
**8**	**In patients with Stage IIB-IIIC TNBC, initial staging investigations** should include imaging of the chest/abdomen, +/− pelvis, and bone.	Strong recommendation ●●●	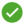	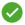
**9**	**For patients with clinical Stage II or III TNBC:** a) **NAC** with taxane plus carboplatin followed by anthracycline plus cyclophosphamide, with pembrolizumab administered from initiation of chemotherapy followed by adjuvant pembrolizumab every 3 weeks for up to 9 cycles is recommended. **b)** **If pembrolizumab is contraindicated**, a neoadjuvant anthracycline- and taxane-based chemotherapy regimen with consideration of including a platinum agent is recommended. **c)** **For patients who did not receive neoadjuvant chemotherapy plus immunotherapy,** adjuvant anthracycline- and taxane-based chemotherapy (e.g., dose-dense AC followed by paclitaxel) is recommended.	Strong recommendation ●●● Strong recommendation ●●● Strong recommendation ●●●	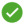 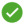 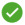	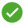 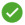 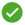
**10**	**For patients with Stage II or III TNBC where a pCR occurs following neoadjuvant pembrolizumab plus chemotherapy,** continuation of adjuvant pembrolizumab every 3 weeks for up to 9 cycles is recommended.	Strong recommendation ●●●	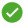	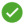
**11**	**In patients who receive neoadjuvant chemotherapy for TNBC and have a high burden of disease post-neoadjuvant therapy (i.e., RCB-III),** repeat staging investigations of chest/abdomen, +/− pelvis, and bone scan, after surgery could be considered.	Expert opinion ○	NC	NC
**12**	**For patients with Stage II–III TNBC who have residual invasive disease following neoadjuvant systemic therapy (with or without immunotherapy),** adjuvant systemic treatment should be guided by germline BRCA status: **a)** **In patients with germline *BRCA1/2* pathogenic variants,** adjuvant olaparib is recommended. **b)** **In patients with germline *BRCA*–wild type disease,** adjuvant capecitabine is recommended. **c)** **In patients who received neoadjuvant pembrolizumab,** continuation of adjuvant pembrolizumab every 3 weeks for up to 9 cycles, with addition of olaparib or capecitabine as above, could be considered.	Strong recommendation ●●● Strong recommendation ●●● Expert opinion ○	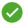 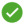 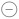 Combination of ICI and olaparib may be considered on an individual basis	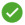 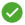 NC
** *Follow-up, surveillance and special populations* **				
**13**	**For patients with TNBC who have completed adjuvant treatment:** a) History and physical examination should be performed every 3–6 months for the first 2 years, every 6 months in the third year, and annually thereafter. Annual mammography, with additional breast imaging as needed based on breast density should also be performed in those with residual breast tissue. Routine imaging, other than breast, in asymptomatic patients is not recommended. **b)** Follow-up assessments can be completed by any trained healthcare provider and should include evaluation for signs and symptoms of recurrence or new ipsilateral/contralateral breast cancers, and monitoring for late effects of prior treatment (e.g., cardiac risk in those treated with anthracyclines, or immune-related adverse events in those who received immunotherapy).	Strong recommendation ●●● Strong recommendation ●●●	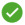 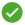	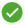 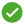
**14**	**For patients with early-stage TNBC of uncommon histologic subtypes,** management should be discussed in a multidisciplinary team setting, including pathology review, to guide a tailored approach to surgery and systemic therapy.	Expert opinion ○	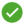	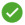

○, Expert opinion; ●●, moderate recommendation; ●●●, strong recommendation; 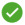, alignment; 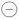, some variation; and NC, not covered.

### 4.1. Stage I Recommendations

**Recommendation 5:** 
*For patients with Stage I TNBC whose disease is node-negative, confirmed by ultrasound or ultrasound-guided FNA prior to surgery, and for situations where the clinical stage of the tumour is as follows:*

(a) *≤1 cm (T1a, b), upfront surgery is the standard of care. [Strong recommendation]*
(b) *>1.0 cm and ≤2 cm (T1c), NAC (without immunotherapy) could be considered [Moderate recommendation]*

For Stage I TNBC, the possibility of overtreatment is an important consideration in clinical decision-making. With respect to the role of neoadjuvant therapy, a large NCDB analysis of more than 35,000 patients with T1N0 TNBC showed that NAC was not associated with improved OS compared with upfront surgery followed by adjuvant chemotherapy in unselected patients [43]. However, among patients with T1c disease who received neoadjuvant therapy, those who experienced pCR had better outcomes, underscoring the prognostic, rather than predictive, value of a complete pathological response [43]. This association was not observed in patients with smaller (T1a-b) tumours. Consistent with these findings, international guidelines recommend upfront surgery for small node-negative TNBC, with ESMO advising surgery for tumours ≤ 1 cm and an ASCO expert review setting a slightly higher threshold of ≤1.5 cm [5,44]. For T1c tumours, NAC could be considered, not because it improves the baseline outcomes across all patients, but because it offers prognostic information through pathologic response and the potential to guide adjuvant escalation in patients with residual disease [40].

With respect to treatment intensification with immunotherapy in Stage I TNBC, it is important to recognize that while neoadjuvant chemo-immunotherapy based on the KEYNOTE-522 regimen is now standard of care for Stage II–III TNBC, the existing evidence does not support routine escalation with immunotherapy in Stage I disease [45]. Most randomized trials evaluating immunotherapy, including KEYNOTE-522, excluded patients with small, node-negative tumours (<2 cm). Given the generally favourable prognosis of stage I TNBC, (particularly for T1a–b tumours), the potential risks of overtreatment, treatment-related toxicity and long-term complications outweigh theoretical benefit of immunotherapy in this setting.

**Recommendation 6:** 
*For patients with Stage I TNBC who undergo upfront surgery and whose tumour is as follows:*

(a) *≤0.5 cm (T1a), adjuvant chemotherapy is not recommended. [Moderate recommendation]*
(b) *>0.5 cm and ≤1.0 cm (T1b), adjuvant chemotherapy could be considered through shared decision-making. [Moderate recommendation]*
(c) *>1.0 cm and ≤2 cm (T1c), adjuvant chemotherapy (without immunotherapy) should be considered. [Moderate recommendation]*

Evidence from observational and retrospective cohort studies consistently demonstrate a size-dependent absolute benefit of adjuvant chemotherapy in Stage I TNBC, with diminishing or absent benefit in smaller tumours (Table 4). The most comprehensive dataset is the Netherlands Cancer Registry analysis [46], which followed 4366 women with Stage I TNBC diagnosed between 2005 and 2015 for a median of 8.2 years. In this cohort, adjuvant chemotherapy was associated with a significant improvement in breast cancer-specific survival (BCSS) in patients with pT1c tumours, but no benefit was observed for pT1b tumours, and the outcomes were worse in the pT1a subgroup. Multiple institutional and registry-based studies support this size-stratified pattern, with consistent benefit observed in pT1c tumours and little or no benefit in pT1a–b disease.

A systematic review and meta-analysis [47] (12 cohorts, nearly 50,000 patients) synthesized these data and concluded that chemotherapy improves OS in patients with both pT1b and pT1c tumours. Importantly, across all studies, no chemotherapy benefit has ever been shown for patients with small pT1a tumours.

International guidelines and consensus statements generally align with the size-stratified evidence but differ in their thresholds and emphasis. ESMO (2024) recommends adjuvant chemotherapy for patients with ≥pT1bN0 disease and not for patients with pT1a tumours [5]. An ASCO expert review supports chemotherapy for tumours <1.5 cm (without precisely specifying if this is referring to tumours > 0.5 cm to <1.5 cm), while advising against adjuvant therapy in Stage I patients who experience a pCR after neoadjuvant treatment, but recommending escalation for those with residual disease [44]. Finally, the St. Gallen 2025 Consensus was more varied: 76% of panellists supported adjuvant chemotherapy for tumours as small as 0.5 cm, though most emphasized tailoring decisions to high-risk features such as grade 3 histology, high Ki-67, young age, or germline *BRCA* mutation [48].

**Table 4:** Evidence for adjuvant chemotherapy in Stage I TNBC.

Study	Data Source/Design	N (Stage I TNBC)	Tumour Size Groups	Key Quantitative Findings	Main Conclusion
Steenbruggen et al., 2020 [46]	Netherlands Cancer Registry; population-based retrospective cohort	**4366**	T1a, T1b, T1c	**BCSS aHR (95% CI):** T1a 4.28 (1.12–16.44); T1b 1.12 (0.51–2.49); T1c 0.60 (0.43–0.82); **OS mirrored BCSS**	Chemotherapy benefit **most evident in T1c**; no benefit in T1b; possible harm in T1a
An et al., 2020 [49]	Single-centre cohort + meta-analysis	**351** (cohort); **1525** (meta-analysis)	T1a, T1b, T1c	**Cohort study** **5-yr RFS HR (95% CI):** T1a HR 3.99 (0.005–317.5); T1b HR 0.64 (0.05–7.74); T1c HR 0.107 (0.047–0.244); **Meta-analysis** **RR HR (95% CI):** T1a 0.64 (0.31–1.33); T1b 0.62 (0.42–0.92); T1c not evaluated	Cohort benefit confined to **T1c**; meta-analysis suggests **recurrence reduction in T1b**, not T1a
Fasano et al., 2022 [50]	Dual-centre U.S. retrospective cohort	**282**	T1a, T1b, T1c	**5-yr OS (with vs. without CT):** T1a 100% vs. 100% (*p* = 0.3778); T1b 100% vs. 95.8% (*p* = 0.2362); T1c 93.2% vs. 75.2% (*p* = 0.008);	OS benefit restricted to **T1c**
Bravo-Solarte et al., 2023 [51]	SEER database retrospective analysis	**1739**	T1a only	**OS HR (95% CI):** T1a 0.63 (0.35–1.13); **BCSS HR (95% CI):** T1a 0.95 (0.37–2.43)	No survival benefit for chemotherapy in **T1a**
Carbajal-Ochoa et al., 2024 [52]	SEER database retrospective analysis	**11,510**	T1b, T1c	**OS HR (95% CI):** T1b 0.52 (0.41–0.68); T1c 0.54 (0.47–0.62); **BCSS HR (95% CI):** T1b 0.70 (0.45–1.07); T1c: 0.79 (0.63–0.99)	Strong support for **T1c**; OS but not significant BCSS benefit in T1b
Moraes et al., 2025 [47]	Systematic review and meta-analysis of retrospective cohorts	**48,371 (32,341 received adjuvant CT)**	T1a, T1b, T1c	**OS HRs:** T1a 0.87 (0.50–1.52); T1b 0.72 (0.56–0.93); T1c 0.72 (0.55–0.94), *p* = 0.01.	OS benefit observed in **T1b and T1c**, but **not in T1a** tumours

**Abbreviations:** CT = chemotherapy; OS = overall survival; BCSS = breast cancer-specific survival; RFS = recurrence-free survival; HR = hazard ratio; aHR = adjusted hazard ratio; RR = risk ratio.

**Recommendation 7:** 
*For patients with Stage I (T1b-T1c, N0) TNBC receiving chemotherapy:*

(a) *Standard therapy should include a taxane (e.g., paclitaxel or docetaxel) [Strong recommendation].*
(b) *To reduce the risk of long-term cardiotoxicity, anthracycline-containing regimens could be avoided for tumours ≤1 cm [Moderate recommendation].*

Taxanes are a foundational component of chemotherapy for early-stage TNBC and are included in all validated regimens used in this setting. Large randomized trials and patient-level meta-analyses demonstrate that the incorporation of a taxane significantly improves disease-free survival (DFS) and OS in early breast cancer, including hormone receptor-negative disease, with no evidence of diminished benefit in TNBC [53,54]. These data support taxane-based therapy as the backbone of chemotherapy for Stage I TNBC.

Anthracycline–taxane combination regimens have demonstrated superior efficacy compared with taxane-only approaches in higher-risk, hormone-receptor-negative disease. The incremental benefit of anthracyclines is well-established by the ABC trial, which demonstrated a significant benefit for anthracycline–taxane combinations over docetaxel and cyclophosphamide (TC) alone [55,56].

Given the well-recognized risks of anthracyclines, including cardiomyopathy and secondary hematologic malignancies [57,58], anthracycline-sparing regimens are appropriate for selected patients, particularly those with tumours ≤ 1 cm, where the risks outweigh the benefits. A population-based analysis of patients treated with NAC and surgery in Ontario demonstrated that, while most patients received an anthracycline, anthracycline-free regimens were not associated with an increased risk of breast cancer mortality [59]. It is important to note that for the TNBC subtype, almost all (93.6%; N = 1513/1617) patients with TNBC received an anthracycline-based NAC regimen. For the 104 (6.4%) patients who did not, there was no increase in the 5-year cumulative risk of mortality, though the numbers are small [59].

ESMO recommends six to eight cycles of chemotherapy for tumours that are T1c, typically comprising an anthracycline followed by a taxane. If an anthracycline-free regimen is warranted, TC for six cycles is an acceptable option [5]. Similarly, ASCO states that anthracycline–taxane chemotherapy is preferred in TNBC, particularly for patients that are deemed to be high risk [60].

### 4.2. Stage II–III Recommendations

**Recommendation 8:** 
*In patients with Stage IIB-IIIC TNBC, initial staging investigations should include imaging of the chest/abdomen, +/− pelvis, and bone [Strong recommendation].*

Accurate initial staging in TNBC is essential to guide both systemic and local treatment decisions. The aggressiveness of TNBC often creates pressure to pursue extensive imaging, yet evidence consistently shows that the risk of occult metastatic disease is meaningfully increased only in Stage IIB–IIIC tumours [61].

Based on the available evidence and international guidelines, REAL Alliance has previously advocated for staging guided by anatomic extent, rather than biological subtype [4]. No new data have challenged this assumption, and we therefore reiterate that chest, abdomen, ± pelvis, and bone imaging should be performed at baseline in patients with Stage IIB–IIIC TNBC. This position is consistent with most international guidelines, which recommend systemic staging for locally advanced or node-positive disease but not for earlier, asymptomatic stages [5,62]. While some older studies have suggested a limited yield from the inclusion of pelvic imaging in staging for breast cancer, more recent data remain limited [63,64].

Although no trials support one imaging modality over another in asymptomatic patients, positron emission tomography–computerized tomography (PET-CT) is an acceptable option that does permit for simultaneous imaging of chest/abdomen/pelvis and bone. It is important, however, that PET/CT be performed before the initiation of systemic therapy. In our 2024 staging publication, REAL Alliance noted that PET-CT identified distant metastases in 23% of Stage IIB and Stage III breast cancer patients, compared to 11% with conventional imaging [4]. The long-term outcome implications are unknown, as upstaging may shift treatment away from curative intent in patients whose metastatic disease would not be identified on conventional imaging. Given the lack of data regarding the possible role of baseline brain imaging and surveillance among patients with Stage IIb or III TNBC, Jerzak et al. are conducting a single arm, multi-centre prospective cohort study (“INSIGHT”; NCT06247449) to evaluate the incidence of asymptomatic brain metastases in this patient population.

As stated in Recommendation 4, timely initiation of therapy in TNBC is key. As such, in health systems where delays are possible due to access issues, the most readily available imaging modality should be prioritized to avoid compromising timely treatment.

**Recommendation 9:** 
*For patients with clinical Stage II or III TNBC:*

(a) *NAC with taxane plus carboplatin followed by anthracycline plus cyclophosphamide, with pembrolizumab administered from initiation of chemotherapy followed by adjuvant pembrolizumab every 3 weeks for up to 9 cycles is recommended [Strong recommendation].*
(b) *If pembrolizumab is contraindicated, a neoadjuvant anthracycline- and taxane-based chemotherapy regimen with consideration of including a platinum agent is recommended [Strong recommendation].*
(c) *For patients who did not receive neoadjuvant chemotherapy plus immunotherapy, adjuvant anthracycline- and taxane-based chemotherapy (e.g., dose-dense AC followed by paclitaxel) is recommended [Strong recommendation].*

The KEYNOTE-522 clinical trial assessing the addition of pembrolizumab to neoadjuvant chemotherapy in TNBC was practice-changing for Stage II–III TNBC patients [45,65]. As the largest and most definitive trial in early TNBC, it demonstrated not only a significant improvement in pCR (+13.6 percentage points; 64.8% vs. 51.2%) but also durable gains in event-free survival (EFS; hazard ratio [HR] 0.63) and, at ~75 months, an OS benefit (5-year OS 86.6% vs. 81.7%; *p* = 0.002) [45,65]. With a clear survival benefit in Stage II–III TNBC, this regimen is the global standard of care [5,62].

REAL Alliance supports the KEYNOTE-522 regimen of chemotherapy + pembrolizumab neoadjuvantly followed by post-operative adjuvant pembrolizumab for 9 further cycles as the current standard of care in eligible patients, but the chemotherapy dosing interval may be nuanced based on patient factors. Although prior trials such as CALGB 9741 [66] and ECOG 1199 [67] established the efficacy of dose-dense anthracycline–taxane schedules and weekly paclitaxel, the KEYNOTE-522 regimen employed weekly paclitaxel with carboplatin, followed by standard-interval (q3-weekly) doxorubicin–cyclophosphamide, rather than a dose-dense approach. In clinical practice, patient frailty, baseline cardiac risk, and institutional capacity may further necessitate individualized chemotherapy scheduling.

Further to the dose scheduling interval, not all TNBC patients are candidates for immune checkpoint inhibition. In patients with contraindications to pembrolizumab or where immunotherapy is not feasible, neoadjuvant chemotherapy alone remains the appropriate alternative standard approach in this setting. In such cases, the incorporation of platinum agents is commonly recommended, given the consistent improvements in pCR observed in trials such as CALGB 40603, GeparSixto, and BrighTNess [68,69,70]. Given the higher relapse risk in Stage II–III disease, most guidelines (ASCO, ESMO) endorse platinum as part of neoadjuvant therapy in this setting. Indeed, the benefit of platinum salts has been seen repeatedly in early TNBC in randomized Phase III trials [71] and in large meta-analyses in terms of pCR (RR 1.44), DFS (HR 0.63 for neoadjuvant platinum, 0.69 for adjuvant platinum), and OS (HR 0.69 for neoadjuvant, 0.70 for adjuvant) [72], and therefore should be offered to those with T2N0 disease or higher. For patients who are unable to tolerate anthracyclines, the NEOPACT trial provides a rational alternative: pembrolizumab with carboplatin and docetaxel achieved a pCR rate of 58% and a 3-year EFS of 86%, outcomes approaching those of KEYNOTE-522 [73]. An ongoing Phase 3 trial (NCI Scarlet; NCT05929768) is evaluating this regimen vs. the standard KEYNOTE-522 protocol.

Finally, a subset of patients with Stage II–III TNBC may not receive neoadjuvant therapy, either due to clinical circumstances or treatment sequencing decisions. In such cases, adjuvant anthracycline- and taxane-based chemotherapy remains the recommended standard, reflecting the established survival benefit of these regimens in high-risk early TNBC. Patients who have had surgery without neoadjuvant chemotherapy and have confirmed pathology of >pT2 and/or node-positive disease should receive adjuvant chemotherapy.

**Recommendation 10:** 
*For patients with Stage II or III TNBC where a pCR occurs following neoadjuvant pembrolizumab plus chemotherapy, continuation of adjuvant pembrolizumab every 3 weeks for up to 9 cycles is recommended [Strong recommendation].*

The continuation of pembrolizumab into the adjuvant setting is supported by guidelines, including those from ASCO and ESMO, and reflects the design of the pivotal KEYNOTE-522 trial, which integrated pembrolizumab across both the neoadjuvant and adjuvant phases [5,45]. REAL Alliance supports this approach for patients with high-risk early TNBC, recognizing the robust, long-term data confirming the survival benefit of this treatment strategy in the overall population [65].

Due to the excellent prognosis of patients who experience pCR with neoadjuvant therapy, there is interest in de-escalating the adjuvant component. As such, de-escalation trials for patients following pCR are underway, including OptimICE-pCR [74]. While clinical trial enrollment is encouraged, formal recommendations with regard to de-escalation according to risk stratification cannot be made until the trial results mature and become available.

**Recommendation 11:** 
*In patients who receive neoadjuvant chemotherapy for TNBC and have a high burden of disease post-neoadjuvant therapy (i.e., RCB-III), repeat staging investigations, of chest/abdomen, +/− pelvis, and bone scan, after surgery could be considered [Expert opinion].*

Residual cancer burden (RCB), a standardized pathological measure of residual disease after neoadjuvant therapy, is a validated prognostic marker in TNBC that refines recurrence risk assessment [75]. Patients with RCB-III are at particularly high risk of early relapse, raising concern that overt metastatic disease may already be present. An exploratory analysis of KEYNOTE-522 showed that higher RCB scores are strongly associated with worse EFS [76].

Although no prospective trials have evaluated routine restaging, repeating imaging after surgery in patients with RCB-III, or with unexpectedly high residual disease, may provide clinically relevant information by identifying distant metastases that could alter subsequent management. REAL Alliance therefore suggests that such restaging be considered in this high-risk subgroup, while recognizing this approach as being expert opinion-driven rather than evidence-based. Consistent incorporation of the RCB score into clinical decision-making may also help to guide adjuvant treatment and surveillance escalation.

**Recommendation 12:** 
*For patients with Stage II–III TNBC who have residual invasive disease following neoadjuvant systemic therapy (with or without immunotherapy), adjuvant systemic treatment should be guided by germline BRCA status:*

(a) *In patients with germline BRCA1/2 pathogenic variants, adjuvant olaparib is recommended [Strong recommendation].*
(b) *In patients with germline BRCA–wild type disease, adjuvant capecitabine is recommended [Strong recommendation].*
(c) *In patients who received neoadjuvant pembrolizumab, continuation of adjuvant pembrolizumab every 3 weeks for up to 9 cycles, with addition of olaparib or capecitabine as above, could be considered [Expert opinion].*

The absence of a pCR (particularly ≥RCB-II) after neoadjuvant therapy is a poor prognostic factor in TNBC and is associated with a substantially increased risk of early recurrence, particularly within the first two to three years [77,78]. As a result, residual invasive disease represents a critical opportunity for post-neoadjuvant treatment escalation.

For patients with germline *BRCA1/2* pathogenic variants and residual disease after neoadjuvant chemotherapy, international guidelines recommend adjuvant olaparib, based on the Phase III OlympiA trial [5,62]. OlympiA enrolled patients with high-risk, HER2-negative early breast cancer who had completed local therapy and (neo)adjuvant chemotherapy; 82% had TNBC. TNBC-specific eligibility included residual invasive disease after NAC or, following upfront surgery, node-positive disease or node-negative tumours ≥pT2 [79]. In the second prespecified interim analysis (median follow-up of 3.5 years), adjuvant olaparib significantly improved invasive DFS with absolute improvements of approximately 7% at 4 years [79]. With longer-term follow-up (median follow-up of 6.1 years), these benefits were maintained, with sustained improvements in invasive DFS (HR 0.65), distant DFS (HR 0.65), and OS (HR 0.72), and no new safety signals [80]. It should be noted that patients in this trial did not receive neoadjuvant pembrolizumab.

For patients without germline *BRCA* mutations, adjuvant capecitabine is supported by the CREATE-X trial, in which TNBC patients with residual disease experienced significant improvements in both DFS and OS [81]. Among 887 HER2-negative patients with residual disease after NAC, the TNBC subgroup (*n* = 286; 32%) experienced significant gains in 5-year DFS (69.8% vs. 56.1%) and OS (78.8% vs. 70.3%) with capecitabine compared with observation, with manageable toxicity. These findings have been confirmed in patient-level meta-analyses and real-world studies and are uniformly endorsed by international guidelines [5,62,82,83,84,85,86]. This strategy remains especially relevant in settings where pembrolizumab is not available, or in patients who are unable to receive immunotherapy.

In patients who received neoadjuvant pembrolizumab, the continuation of adjuvant pembrolizumab every 3 weeks up to 9 cycles is consistent with the KEYNOTE-522 trial design and current guideline recommendations. However, patients with residual invasive disease—particularly those with a high RCB—remain at substantial risk of early recurrence despite immunotherapy, underscoring the need for post-neoadjuvant treatment intensification [76]. Although capecitabine and olaparib have not been prospectively studied in combination with pembrolizumab in early-stage TNBC, the available data from metastatic disease and early-phase studies suggest that these combinations are feasible and do not introduce unexpected safety concerns [87,88,89,90,91]. However, the magnitude of the benefit, if any, in the post-neoadjuvant setting remains unknown, and there is currently no prospective evidence to guide the treatment selection, sequencing, or combination with pembrolizumab.

The recommendations for combination or sequencing strategies are therefore based on the extrapolation of results from the metastatic setting, including the KEYLYNK-009 study, which did not meet its primary efficacy endpoint but did support the safety of the combination of a PARP inhibitor and PD-1/PD-L1 combination therapy [91]. In the absence of prospective data, REAL Alliance supports continuation of pembrolizumab with consideration given to the addition of olaparib in germline *BRCA*-mutated disease or capecitabine in *BRCA*–wild type disease. It should be noted that olaparib should not be combined with capecitabine, due to overlapping hematologic and gastrointestinal toxicities.

### 4.3. Follow-Up, Surveillance and Special Populations

**Recommendation 13:** 
*For patients with TNBC who have completed adjuvant treatment:*

(a) *History and physical examination should be performed every 3–6 months for the first 2 years, every 6 months in the third year, and annually thereafter. Annual mammography, with additional breast imaging as needed based on breast density, should also be performed in those with residual breast tissue. Routine imaging, other than breast, in asymptomatic patients is not recommended [Strong recommendation].*
(b) *Follow-up assessments can be completed by any trained healthcare provider and should include evaluation for signs and symptoms of recurrence or new ipsilateral/contralateral breast cancers, and monitoring for late effects of prior treatment (e.g., cardiac risk in those treated with anthracyclines, or immune-related adverse events in those who received immunotherapy) [Strong recommendation].*

Patients with TNBC face the greatest risk of recurrence in the first two to three years following treatment completion, with multiple prospective cohorts confirming this early relapse pattern [92]. This biology supports close surveillance in the first 2–3 years after treatment, followed by a stepwise de-escalation of the intensity of surveillance as recurrence risk declines—an approach that not only matches the disease dynamics but also has important implications for workload management in our health system and supporting patients’ return to routine life and long-term survivorship. Regular history and physical examination remain the cornerstone of follow-up, with annual mammography. Any supplemental breast imaging should be guided by breast density and clinical/radiological suspicion. From a surveillance perspective for metastatic disease, investigations should be based on suspicious symptoms or signs identified by the patient and provider and should be targeted to address the symptoms and signs. It has been shown that routine “surveillance” body imaging and bloodwork does cause more harm than benefit, with no improvement in survival but increased iatrogenic complications. As such, it is important to note that in the adjuvant setting, neither routine imaging nor laboratory tests are indicated in asymptomatic patients.

Long-term toxicity surveillance is increasingly important due to the complexity of treatment and improved prognosis overall. Patients exposed to anthracyclines require ongoing awareness of potential late-onset cardiomyopathy. The European Society of Cardiology (ESC) cardio-oncology consensus guidelines recommend baseline and periodic echocardiography [93]. For those who received immune checkpoint inhibitors, vigilance for immune-related adverse events is required well beyond the end of treatment, as late toxicities can emerge months later and involve multiple organ systems [94]. Ensuring that primary care practitioners (PCPs) are well-informed of these potential late effects of treatment is critical and improved communication strategies and resources to aid in the identification and management of late effects, both known and emergent, will be critical in the future.

Patients with germline *BRCA1/2* pathogenic variants require additional hereditary cancer surveillance and counselling, including consideration of risk-reducing strategies, as outlined in the ESMO guideline on hereditary breast cancer and risk-reducing surgery [26].

With regard to the type of healthcare provider involved in the delivery of follow-up care, there have been many randomized and cohort-based studies completed assessing the role of PCP and oncologist in the delivery of post-treatment care for breast cancer survivors over several decades. This work has demonstrated the same medical outcomes and quality of life but better patient satisfaction and a lower cost with PCP follow-up compared to specialist follow-up clinics [95]. The implementation of this strategy is dependent on the availability of PCPs to the patient, and on appropriate communication and knowledge transfer between oncologist and PCP teams.

**Recommendation 14:** 
*For patients with early-stage TNBC of uncommon histologic subtypes, management should be discussed in a multidisciplinary team setting, including pathology review, to guide a tailored approach to surgery and systemic therapy [Expert opinion].*

TNBC is heterogeneous, and several rare histologies differ in prognosis and chemotherapy sensitivity [96]. For example, apocrine (androgen receptor-positive) TNBC often shows a limited response to standard chemotherapy, yet a more indolent course overall, supporting the consideration of adjustment in the standard treatment approach in carefully selected cases. Conversely, high-grade metaplastic carcinoma (a term that is also inconsistently applied) has inferior outcomes and more variable chemo-sensitivity.

Since rare histological subtypes are uncommon and prone to misclassification, a multidisciplinary team (MDT) review with formal pathology re-evaluation is critical to avoid over- or under-treatment. Multiple studies have demonstrated improvements in survival outcomes with structured, specialist MDTs for breast cancer [97]. Similarly, in a >16,000-patient cohort from Fudan, survival was superior when patients were managed by a well-organized MDT compared with a disorganized or no MDT, underscoring that structure and attendance matter [98]. These studies included patients with more common histologies but underscored the impact and importance of MDT.

Major guidelines endorse individualized management for special histologies and emphasize multidisciplinary decision-making in early breast cancer care [5].

## 5. Metastatic TNBC

**Figure 3:**
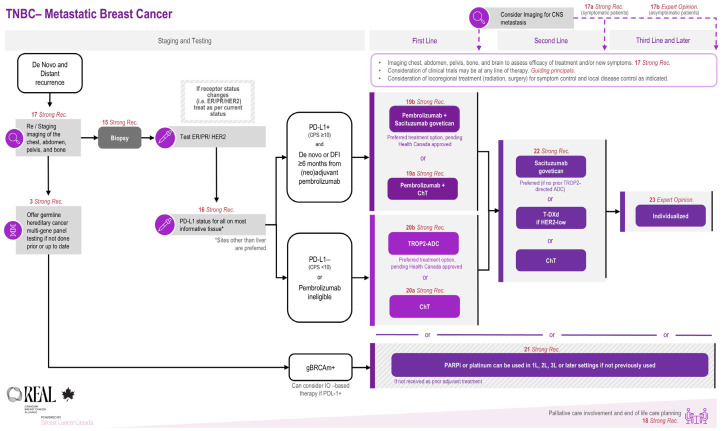
**REAL Alliance recommendations for the management of TNBC in the metastatic setting.** Abbreviations: 1 L = first-line; 2 L = second line; 3 L+ = third line and later; ADC = antibody–drug conjugate; CNS = central nervous system; CPS = combined positive score; ChT = chemotherapy; DFI = disease-free interval; ER = Estrogen Receptor; gBRCA1/2 = germline BRCA1/2 pathogenic variant; HER2 = Human Epidermal Growth Factor Receptor 2; HER2-low = human epidermal growth factor receptor 2 low expression (IHC 1+ or IHC 2+/ISH−); ICI = immune checkpoint inhibitor; IHC = immunohistochemistry; IO = immune-oncology; ISH = in situ hybridization; PARPi = poly(ADP-ribose) polymerase inhibitor; PD-L1 = programmed death-ligand 1; PR = Progesterone Receptor; Rec. = recommendation; T-DXd = trastuzumab deruxtecan; TNBC = metastatic triple-negative breast cancer; TROP2 = trophoblast cell-surface antigen 2; TROP2-ADC = TROP2-directed antibody–drug conjugate; and wtBRCA1/2 = BRCA1/2 wild-type.

The prevalence and pattern of the development of metastatic disease in TNBC is unique. In general, it is estimated that up to 30–40% of patients diagnosed with early-stage TNBC may go on to experience a recurrence or distant metastases. The timing of the development of a metastatic disease is typically earlier than in other disease subtypes, with the majority occurring within the first two to three years of completion of adjuvant therapy. Given the aggressive nature of this disease, roughly 30–50% of patients with metastatic TNBC will not reach second-line therapy [99]. However, new data—particularly with antibody drug conjugates (ADCs)—have begun to redefine treatment practice in metastatic TNBC. This section outlines REAL Alliance recommendations for systemic therapy in metastatic TNBC, organized by key clinical decision points, including baseline assessment, first-line treatment, and subsequent lines of therapy (Table 5). A summary treatment algorithm is provided in Figure 3.

### 5.1. Considerations in Metastatic Setting

**Recommendation 15:** 
*As consistently endorsed by REAL Alliance across all breast cancer subtypes, biopsy of a recurrent lesion(s) should be considered at the time of diagnosis to evaluate biomarkers or confirm the diagnosis [Strong recommendation].*

REAL Alliance strongly recommends a biopsy at first relapse across all breast cancer subtypes (HER2-positive, hormone receptor-positive, TNBC) to confirm recurrence and reassess biomarkers (ER, PR, HER2) [3]. Many prospective studies have demonstrated a significant impact on patient care with re-biopsy at the time of metastatic disease diagnosis [104]. This practice reflects the biological heterogeneity of breast cancer, where the receptor status may change at relapse, and it is consistent with the standard of care across all subtypes of breast cancer. If the receptor status differs from the primary tumour, subsequent systemic treatment should be guided by the current receptor status.

**Recommendation 16:** 
For all patients with advanced/metastatic TNBC, the tumour’s PD-L1 status should be determined on the most informative tissue sample*, if not previously tested or if the prior result was negative [Strong recommendation]. (* Expression of PD-L1 may be lower in liver metastases, therefore sites other than the liver are preferred as they will yield more informative results [105]).

In metastatic TNBC, re-biopsy is also essential to evaluate the PD-L1 status (provided there is not a previous positive result), to ensure appropriate and the most effective systemic therapy. The landmark trial KEYNOTE-355 trial [106] showed that adding a PD-L1 inhibitor to chemotherapy significantly improves outcomes in patients with PD-L1-positive tumours in the first-line setting (combined positive score [CPS ≥ 10]). Since 2020, pembrolizumab plus chemotherapy has become the first-line standard for these patients, as endorsed by all major guidelines. Biopsies from soft tissue, lung, or lymph node metastases provide the most dependable results. By contrast, liver lesions often yield falsely low PD-L1 [105,107]. Bone biopsies demonstrate lower rates of PD-L1 positivity than other sites of metastasis [105,107], as antigenic epitopes are impacted by decalcification during processing. A negative PD-L1 result from these sites should therefore be interpreted with caution and, where possible, be confirmed by biopsy from a more suitable metastatic lesion. CPS should be tested with an appropriate and validated assay [107]. Communication and collaboration between pathologists and medical oncology is key to ensure that optimal treatment is offered.

**Recommendation 17:** 
*For patients with a new diagnosis of metastatic TNBC:*

(a) *Comprehensive re-staging with imaging of the chest, abdomen, pelvis, and bone. Brain imaging is also recommended in patients with symptoms that are suggestive of CNS involvement [Strong recommendation].*
(b) *Baseline brain imaging may also be considered in asymptomatic patients, with repeat assessment at the time of disease progression [Expert opinion].*

This position is consistent with previous REAL Alliance consensus statements in staging and in HER2+ disease [3,4]. Assessment of the extent of metastatic disease enables a more accurate evaluation of the disease response and prognostication. For TNBC specifically, the risk of CNS metastases is approximately 25–46% [108,109]. This heightened risk raises ongoing uncertainty regarding whether earlier detection of CNS disease—before the onset of neurologic symptoms—could meaningfully alter the outcomes in selected patients [108,109]. Earlier identification of limited CNS disease may allow for multidisciplinary management, including consideration of CNS-directed local therapies in selected patients. Accordingly, while guidelines endorse brain imaging for patients with neurological symptoms, they are more cautious when it comes to asymptomatic patients [6,62,110].

Evidence to support routine CNS surveillance in asymptomatic TNBC is sparse and based on observational data [111,112]. In a single-centre cohort from Korea, screening MRI identified asymptomatic brain metastases in 24% of patients with metastatic TNBC [113]. While improved survival was observed (23.7 vs. 7.3 months) in surveillance-detected cases compared to symptomatic patients, the contribution of the lead-time bias versus the meaningful clinical benefit remains uncertain. This uncertainty is particularly relevant given the morbidity associated with CNS-directed local therapies. Treatments that are commonly employed after detection carry well-documented risks (i.e., long-term neurocognitive decline and radiation necrosis) that must be considered when evaluating the value of routine surveillance [114,115,116,117]. Therefore, the absence of randomized trial evidence creates clinical equipoise with regard to routine imaging of the brain in asymptomatic patients and underscores the importance of shared decision-making, balancing risks and benefits. REAL Alliance explicitly categorizes its recommendation for baseline and progression brain MRI in asymptomatic TNBC as an expert opinion. This reflects the panel’s judgement that, in selected patients, early detection of CNS disease may meaningfully influence management and outcomes. The Canadian SYMptom trial may help to resolve these uncertainties; the final analysis after 18 months follow-up is pending [118]. In the meantime, MDT discussions are essential to optimize patient care.

**Recommendation 18:** 
*Early discussion about goals of care and palliative support should be encouraged for patients with metastatic disease to ensure that treatment aligns with patients’ values and optimizes their quality of life [Strong recommendation].*

Randomized control trial evidence supports early palliative involvement to improve outcomes in patients with advanced cancer [119]. In mixed solid-tumour populations, meta-analyses confirm benefits in symptom burden, patient-reported quality of life, and care, consistent with patient values [120,121]. Breast cancer-specific randomized data also show that structured palliative interventions significantly increase high-quality goals-of-care discussions without worsening mood or function [122]. Reflecting this evidence, ASCO recommends that early, concurrent palliative care resources be initiated for all patients with advanced cancer [101]. On this basis, early goals-of-care and palliative discussions should be encouraged across metastatic breast cancer—including HER2-positive, hormone receptor-positive, and TNBC—to ensure that treatment aligns with patients’ values and their quality-of-life priorities.

### 5.2. First-Line Setting

**Recommendation 19:** 
*For patients with metastatic TNBC in the first-line setting whose tumour is PD-L1-positive (CPS ≥ 10):*

(a) *First-line pembrolizumab in combination with chemotherapy (paclitaxel, nab-paclitaxel, or carboplatin/gemcitabine) is currently recommended [Strong recommendation].*
(b) Pembrolizumab in combination with sacituzumab govitecan can be considered and may be preferred* [Strong recommendation]. (* Pending Health Canada approval).

Pembrolizumab combined with chemotherapy is a well-established first-line standard in PD-L1-positive metastatic TNBC, in patients who have no contraindication to immunotherapy, supported by the pivotal KEYNOTE-355 trial. In patients with CPS ≥ 10 tumours, adding pembrolizumab significantly improved outcomes, with a median PFS of 9.7 vs. 5.6 months (HR ≈ 0.65) and median OS of 23.0 vs. 16.1 months (HR ≈ 0.73) compared with chemotherapy alone [45]. This trial included patients with a disease-free interval (DFI) of ≥6 months after chemotherapy or ≥12 months after prior PD-1/PD-L1 therapy. The ESMO guidelines therefore recommend first-line pembrolizumab in PD-L1-positive patients with a DFI of ≥6 months [100]. Other PD-L1 inhibitors such as atezolizumab were also investigated, but inconsistent results across studies led to withdrawal of the indication in TNBC in the U.S, leaving pembrolizumab as the preferred agent in most guidelines [123,124,125,126,127].

With pembrolizumab now established as part of first-line therapy, the optimal partner backbone (chemotherapy agent or otherwise) is now the focus of clinical trials. Sacituzumab govitecan, an ADC targeting trophoblastic cell-surface antigen 2 (TROP-2), a cell-surface glycoprotein overexpressed in ~80% of TNBC tumours, with a topoisomerase I payload, has already demonstrated survival benefit in later-line disease [128,129,130,131]. Initial results of the Phase III ASCENT-04/KEYNOTE-D19 have been reported, showing that combining sacituzumab govitecan with pembrolizumab in PD-L1-positive, first-line TNBC improved outcomes compared with chemo–pembrolizumab, with a median PFS of 11.2 vs. 7.8 months (HR ≈ 0.65) and extended the median duration of response (16.5 vs. 9.2 months), with manageable toxicity [132]. ADC-induced tumour cell death may promote antigen release, dendritic cell maturation, and T-cell activation, potentially enhancing tumour immunogenicity and synergy with immune checkpoint inhibition, such as PD-1 blockade [133]. Importantly, patient-reported outcomes presented at ESMO 2025 demonstrated that sacituzumab govitecan plus pembrolizumab did not compromise overall quality of life and improved several functional domains—including emotional functioning and pain—relative to chemo–pembrolizumab combinations [134]. While OS results are immature (and may be affected by crossover in the ASCENT-04 trial), given the high proportion of metastatic TNBC patients who do not ultimately receive second- or later-line therapies, first-line use of ADC therapy likely has an important clinical role. The promise of immunotherapy–ADC combinations also extends beyond sacituzumab govitecan.

REAL Alliance endorses pembrolizumab plus chemotherapy as the standard for PD-L1-positive, metastatic TNBC. However, the panel also highlights the compelling ASCENT-04 results, in which sacituzumab govitecan plus pembrolizumab outperformed chemo–pembrolizumab with meaningful improvements in PFS and the duration of response. Given the magnitude and consistency of benefit, the panel considers sacituzumab govitecan + pembrolizumab the preferred option, pending Health Canada approval, especially as the clinical trial data mature.

**Recommendation 20:** 
*For patients with locally recurrent, unresectable, or metastatic TNBC in the first-line setting whose tumours are PD-L1-negative (CPS < 10) or for whom immunotherapy is contraindicated:*

(a) *First-line chemotherapy is recommended, with regimen choice guided by prior treatments, disease characteristics, disease-free interval, and patient preferences, incorporating shared decision-making [Strong recommendation].*
(b) A TROP-2-directed ADC may be considered as the preferred option in this setting* with shared decision-making regarding efficacy and safety profiles [Strong recommendation] (* Pending Health Canada approval).

For patients with PD-L1-negative tumours or those relapsing soon after prior perioperative immunotherapy exposure (e.g., KEYNOTE-522 regimen), or within the first 6 months on immunotherapy, metastatic TNBC represents one of the most difficult clinical scenarios [45,62,135].

In this context, Phase III data now support TROP-2-directed ADCs in this setting. In ASCENT-03, which required a DFI ≥6 months after the completion of curative-intent therapy, sacituzumab govitecan significantly improved PFS over physician’s-choice chemotherapy in previously untreated, locally advanced or metastatic TNBC whose tumours were PD-L1-negative or who were not candidates for immunotherapy due to previous use or coexisting medical conditions. Median PFS by blinded independent central review (BICR) was 9.7 vs. 6.9 months (HR 0.62; 95% CI 0.50–0.77; *p* < 0.001), with similar ORR (48% vs. 46%) but a substantially longer median duration of response (12.2 vs. 7.2 months) and a manageable toxicity profile [136]. The OS results in ASCENT-03 are not yet mature, and the study was not powered to detect an OS difference; moreover, the permitted crossover to second-line sacituzumab govitecan in the control arm is expected to dilute any potential between-arm OS signal.

TROPION-Breast02 provides complementary, practice-changing evidence for Dato-DXd in a similar first-line, immuno-ineligible population [137]. Unlike ASCENT-03, TROPION-Breast02 did not require a minimum DFI, allowing for the enrollment of patients regardless of the time since completion of curative-intent therapy—including those with earlier relapse and progression while on immunotherapy or adjuvant chemotherapy. In this Phase III trial, Dato-DXd significantly improved both dual primary endpoints—PFS and OS—versus investigator’s-choice single-agent chemotherapy in patients with locally recurrent inoperable or metastatic TNBC for whom immunotherapy was not an option. The median PFS by BICR was doubled at 10.8 vs. 5.6 months (HR 0.57; 95% CI 0.47–0.69; *p* < 0.0001), with a confirmed ORR of 62.5% vs. 29.3% and median duration of response of 12.3 vs. 7.1 months [137]. The median OS was 23.7 vs. 18.7 months (HR 0.79; 95% CI 0.64–0.98), although importantly, crossover was not allowed in this trial and therefore many patients were unable to subsequently access an ADC. This benefit was consistent across prespecified subgroups, including de novo disease and short DFI (0–12 months), and grade ≥ 3 toxicity rates were in line with chemotherapy despite a markedly longer treatment duration.

Since both sacituzumab govitecan and Dato-DXd have similar PFS benefit, toxicity profiles may become a decisive factor in first-line treatment selection, though in the case of DFI ≤6 months, Dato-DXd would be preferred based on trial design, as noted above. In ASCENT-03, grade ≥ 3 treatment-related adverse events (AEs) occurred in 61% of patients receiving sacituzumab govitecan, driven primarily by neutropenia and gastrointestinal toxicity, whereas in TROPION-Breast02, grade ≥ 3 events occurred in 33% with Dato-DXd, with notably less myelosuppression but higher rates of stomatitis and ocular surface toxicity. Ultimately, the emergence of these ADCs expands options in a setting that has been long characterized by therapeutic scarcity, but careful patient selection, attention to safety profiles, and alignment with patients’ goals remain central to integrating these agents alongside established chemotherapy standards.

**Recommendation 21:** 
*For patients with metastatic TNBC who have a germline BRCA mutation, incorporation of treatment with a PARP inhibitor (or platinum salts), in later lines of therapy or at any point in those with PD-L1-negative disease, is recommended [Strong recommendation].*

Patients with germline BRCA mutations represent a biologically distinct subset of metastatic TNBC, for whom targeted therapy with PARP inhibitors has emerged as a key treatment strategy. The use of olaparib and talazoparib is supported by high-level evidence from the OlympiAD [138] and EMBRACA trials [139], respectively, with both demonstrating superior PFS compared with standard but non-platinum chemotherapy. Notably, these trials enrolled patients across multiple lines of metastatic therapy, whereas OlympiAD included patients who had received no more than two prior chemotherapy regimens for metastatic disease, while EMBRACA enrolled a more heavily pretreated population. As a result, international guidelines, including those from ESMO and ASCO, strongly recommend PARP inhibitor therapy in this population [6,140]. In the absence of PARP inhibitor therapy, platinum-based chemotherapy also plays a role in this population. Evidence from the TNT trial [57] supported by meta-analysis [141] and real-world data [142] indicates improved outcomes with carboplatin over non-platinum regimens in *BRCA*-mutated patients. Reflecting this, ESMO provides cautious guidance, recognizing platinum as an acceptable option but emphasizing the need to balance its use against alternatives such as PARP inhibitors, which demonstrate strong activity and a favourable toxicity profile.

In clinical practice, PARP inhibitors have traditionally been prioritized in PD-L1-negative BRCA mutated patients, where immunotherapy is less effective, although this sequencing preference is based on clinical rationale, rather than trial-defined lines of therapy. As noted in recommendation #3, upfront germline BRCA testing with a multi-gene panel is recommended in patients diagnosed with de novo metastatic TNBC.

### 5.3. Second Line and Beyond

**Recommendation 22:** 
*For patients with metastatic TNBC whose disease has progressed on first-line systemic therapy, sacituzumab govitecan is recommended, if TROP-2-directed ADC was not previously used [Strong recommendation].*

Based on the practice-changing ASCENT trial, sacituzumab govitecan is the preferred therapy beyond the first line for metastatic TNBC [143]. In this Phase III study, sacituzumab govitecan significantly improved both PFS (median 5.6 vs. 1.7 months) and OS (median 12.1 vs. 6.7 months) compared with physician’s-choice chemotherapy (HR 0.48; *p* < 0.001). Although grade ≥ 3 toxicities—most commonly neutropenia and diarrhea—were more frequent with sacituzumab govitecan (51% vs. 33%), AEs were manageable with supportive care, and no treatment-related deaths were reported. As treatment-related deaths due to infections have been reported in the first-line trials, appropriate neutropenia monitoring and prophylaxis is warranted.

As more patients receive TROP-2-directed ADCs earlier in the metastatic course, an increasing proportion will experience disease progression after prior ADC exposure, raising important questions about optimal sequencing. In HER2-low disease, T-DXd represents an alternative ADC option supported by DESTINY-Breast04, which demonstrated a numerical benefit in the TNBC subgroup (58 patients with TNBC overall; ~40 treated with T-DXd) [144]. However, both sacituzumab govitecan and T-DXd deliver a topoisomerase I inhibitor payload, raising concern for cross-resistance. Emerging real-world and retrospective data suggest that sequential use of ADCs with shared payloads following the development of resistance yields no significant benefit [145]. In the largest real-world cohort to date (*n* = 331) of HER2-low MBC treated with sequential sacituzumab govitecan and T-DXd, outcomes with the second topoisomerase I inhibitor ADC were poor: the median PFS2 was 2.6 months, and ~60% progressed at the first assessment. The primary resistance increased from 25.4% on ADC1 to 65.6% on ADC2, including 63.3% of patients who had not been primarily resistant to ADC1, supporting substantial cross-resistance [146].

When both sacituzumab govitecan and T-DXd are viable options in ADC-naïve, chemotherapy pre-treated HER2-low TNBC, head-to-head data are currently lacking, and the guidelines continue to prioritize sacituzumab govitecan due to its robust, TNBC-specific Phase III evidence base. However, T-DXd can be considered in select situations based on the presence of brain metastases, given a large body of evidence demonstrating intracranial activity of T-DXd. Additional investigation will be required to determine if any patients could benefit from sequential topoisomerase I inhibitor ADC following treatment failure.

**Recommendation 23:** 
*For patients with metastatic TNBC whose disease has progressed after second-line therapy, selection of subsequent therapy should be based on prior treatments received in the metastatic setting [Expert opinion].*

For treatment in the third line and beyond, the evidence supporting further therapy is varied. REAL Alliance supports enrollment in clinical trials based on eligibility criteria. Certainly, prioritizing utilization of ADC therapy if options have not been exhausted may have better outcomes than standard chemotherapy, but this must be weighed with toxicity and an incremental expected benefit in the third line and beyond setting. The overall recommendation of REAL Alliance is that further therapy be guided both by the patient’s goals of care, performance status, and predicted tolerability of subsequent therapies. The treatment selection should also consider toxicity profiles and routes of administration. For example, oral agents such as capecitabine may be preferred for convenience and tolerability, while intravenous options such as eribulin may be better suited for patients with prior resistance to taxanes or anthracyclines. Indeed, the paucity of evidence in this setting is reflected in the recommendations of ASCO and ESMO, who do not provide a definitive roadmap for sequencing therapies in metastatic TNBC in the third line and beyond.

## 6. Limitations

This consensus document represents a collaborative evidence-informed effort, but, as with any guidance document, it does have limitations. The supporting literature review was intentionally targeted, rather than a true systematic literature search approach with emphasis placed on pivotal randomized trials, major meta-analyses, and large observational studies that were considered to be the most relevant to contemporary clinical practice. This approach was utilized to provide more timely synthesis of the rapidly evolving evidence in TNBC and to support expert panel deliberation within the modified Delphi framework. We acknowledge that this targeted review could introduce the potential for study-selection bias, whereby relevant studies may be inadvertently omitted, and certain types of evidence may be over-represented. In addition, reliance on randomized trials may emphasize relative treatment effects without fully capturing the absolute benefit in diverse patient populations or the overall impact on quality of life. In several areas covered in this consensus, there indeed is a lack of randomized controlled data requiring the assessment of observational studies which may be subject to residual confounding and treatment selection bias. The process for identifying the ideal pathway of care and consensus statements to improve the consistency of TNBC care across Canada was driven by the subject matter experts within REAL Alliance, independently from the initial literature search. Data from the literature helped to support or refute the subject matter expert recommendations, and as a final step, they were compared to other international guidance documents for consistency. These strategies helped to mitigate any study-selection bias. Finally, as the therapeutic landscape in TNBC continues to evolve rapidly, emerging data from ongoing trials may influence future recommendations. These considerations underscore the importance of regularly updating the REAL Alliance recommendations, which will be reviewed and updated on an annual basis.

## 7. Conclusions

The management of TNBC in both the early-stage and metastatic settings is complex and rapidly evolving. These consensus recommendations from REAL Alliance serve to promote best practices in alignment with the latest evidence and other international guidelines and help to ensure timely and consistent care for patients with TNBC across Canada.

## Figures and Tables

**Table 1:** Summary of REAL Alliance recommendations for general treatment approach with TNBC.

Recommendations for General Treatment Approach with TNBC		REAL	ESMO [5,6]	ASCO [7,8,9]
**1**	**For all aspects of care for patients with TNBC, shared decision-making in partnership with patients and their caregivers is essential.** All subsequent recommendations should be applied within the context of the patient’s preferences, values, and goals, ensuring a collaborative approach to treatment planning.	Expert opinion ○	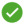	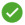
**2**	**Breast cancers with low hormone receptor expression, specifically ER ≤ 10%, PR ≤10%, and lack of HER2 overexpression or gene amplification** (IHC ≤1+ or 2+ with negative ISH) should preferentially be treated as TNBC.	Moderate recommendation ●●	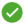	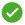
**3**	**Germline hereditary cancer multi-gene panel testing should be offered** to all patients with a diagnosis of TNBC, regardless of age, stage, or family history.	Strong recommendation ●●●	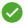	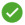
**4**	**Patients with early-stage TNBC should receive initial treatment** (surgery or systemic neoadjuvant therapy) as quickly as possible, ideally within 30 days of biopsy result, recognizing that timely treatment initiation is particularly important, given the aggressive biology of TNBC.	Strong recommendation ●●●	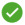	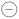 No specific timeframe

○, expert opinion; ●●, moderate recommendation; ●●●, strong recommendation; 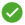, alignment; and 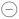, some variation.

**Table 5:** REAL Alliance recommendations for treatment of metastatic TNBC.

Recommendations for metastatic systemic treatment of TNBC		REAL	ESMO [6,100]	ASCO [41,101,102,103]
*Considerations in metastatic setting*				
**15**	**As consistently endorsed by REAL Alliance across all breast cancer subtypes, biopsy of a recurrent lesion(s)** should be considered at the time of diagnosis to evaluate biomarkers or confirm the diagnosis.	Strong recommendation ●●●	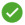	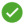
**16**	For all patients with advanced/metastatic TNBC, the tumour’s PD-L1 status should be determined on the most informative tissue sample *, if not previously tested or if the prior result was negative. * Expression of PD-L1 may be lower in liver metastases; therefore sites other than the liver are preferred, as they will yield more informative results.	Strong recommendation ●●●	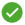	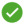
**17**	**For patients with a new diagnosis of metastatic TNBC:** a) Comprehensive re-staging with imaging of the chest, abdomen, pelvis, and bone. Brain imaging is also recommended in patients with symptoms that are suggestive of CNS involvement. b) Baseline brain imaging may also be considered in asymptomatic patients, with repeat assessment at the time of disease progression.	Strong recommendation ●●● Expert opinion ○	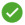 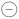 If baseline CNS will change therapy choice	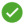 NC
**18**	**Early discussion about goals of care and palliative support** should be encouraged for patients with metastatic disease to ensure that treatment aligns with patients’ values and optimizes their quality of life.	Strong recommendation ●●●	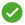	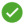
** *First-line* **				
**19**	**For patients with metastatic TNBC in the first-line setting whose tumour is PD-L1-positive (CPS ≥10):** a) First-line pembrolizumab in combination with chemotherapy (paclitaxel, nab-paclitaxel, or carboplatin/gemcitabine) is currently recommended. b) Pembrolizumab in combination with sacituzumab govitecan can be considered and may be preferred * * Pending Health Canada approval.	Strong recommendation ●●● Strong recommendation ●●●	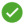 NC	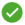 NC
**20**	**For patients with locally recurrent, unresectable, or metastatic TNBC in the first-line setting whose tumours are PD-L1-negative (CPS <10) or for whom immunotherapy is contraindicated:** a) First-line chemotherapy is recommended, with regimen choice guided by prior treatments, disease characteristics, disease-free interval, and patient preferences, incorporating shared decision-making. b) A TROP-2-directed ADC may be considered as the preferred option in this setting * with shared decision-making regarding efficacy and safety profiles. * Pending Health Canada approval	Strong recommendation ●●● Strong recommendation ●●●	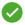 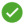	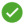 NC
**21**	**For patients with metastatic TNBC who have a germline *BRCA* mutation,** incorporation of treatment with a PARP inhibitor (or platinum salts), in later lines of therapy or at any point in those with PD-L1-negative disease, is recommended.	Strong recommendation ●●●	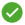	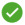
** *Second line and beyond* **				
**22**	**For patients with metastatic TNBC whose disease has progressed on first-line systemic therapy, sacituzumab govitecan is recommended if TROP-2-directed ADC was not previously used.**	Strong recommendation ●●●	NC	NC
**23**	**For patients with metastatic TNBC whose disease has progressed after second-line therapy, selection of subsequent therapy should be based on prior treatments received in the metastatic setting.**	Expert opinion ○	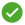	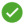

○, Expert opinion; ●●●, strong recommendation; 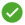, alignment; 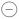, some variation; and NC, not covered.

## Data Availability

No new data were generated.

## Supplementary Materials

The following supporting information can be downloaded at: https://www.mdpi.com/article/10.3390/curroncol33050243/s1. Table S1: Literature search strategy; Table S2: Voting Results.

## Abbreviations

The following abbreviations are used in this manuscript:

AC	Doxorubicin–cyclophosphamide chemotherapy regimen
ADC	Antibody–drug conjugate
ADJ	Adjuvant
AE	Adverse event
aHR	Adjusted hazard ratio
AI	Aromatase inhibitor
AJCC	American Joint Committee on Cancer
ASCO	American Society of Clinical Oncology
BCSS	Breast cancer-specific survival
BICR	Blinded independent central review
BRCA	Breast cancer susceptibility gene
CAP	College of American Pathologists
Cb	Carboplatin
CDK4/6	Cyclin-dependent kinases 4 and 6
ChT	Chemotherapy
CI	Confidence interval
CIHI	Canadian Institute of Health Information
CNS	Central nervous system
CPC	Clinical practice committee
CPS	Combined positive score
cN	Clinical nodal stage (AJCC 8th edition)
cT	Clinical tumour stage (AJCC 8th edition)
Dato-DXd	Datopotamab deruxtecan
DFS	Disease-free survival
DFI	Disease-free interval
EFS	Event-free survival
ER	Estrogen receptor
ESC	European Society of Cardiology
ESMO	European Society for Medical Oncology
ET	Endocrine therapy
FNA	Fine-needle aspiration
gBRCA1/2m+	Germline BRCA1/2 pathogenic variant
HER2	Human epidermal growth factor receptor 2
HER2-low	HER2 low expression (IHC 1+ or IHC 2+/ISH–)
HR	Hazard ratio
IHC	Immunohistochemistry
ICI	Immune checkpoint inhibitor
ISH	In situ hybridization
Ki-67	Ki-67 proliferation index
MDT	Multidisciplinary team
N+	Node-positive
NAC	Neoadjuvant chemotherapy
NCDB	National Cancer Database
NCCN	National Comprehensive Cancer Network
ORR	Objective response rate
OS	Overall survival
PARP/PARPi	Poly(ADP-ribose) polymerase/PARP inhibitor
pCR	Pathologic complete response
PD-L1	Programmed death-ligand 1
PET-CT	Positron emission tomography–computed tomography
PFS	Progression-free survival
PR	Progesterone receptor
Q3W	Every 3 weeks
RCB	Residual cancer burden
Rec.	Recommendation
REAL Alliance	Research Excellence, Active Leadership Canadian Breast Cancer Alliance
RFS	Recurrence-free survival
RR	Risk ratio
LRRT	Locoregional radiotherapy
SABCS	San Antonio Breast Cancer Symposium
SEER	Surveillance, Epidemiology, and End Results Program
T-DXd	Trastuzumab deruxtecan
TAM	Tamoxifen
TC	Docetaxel and cyclophosphamide
TNBC	Triple-negative breast cancer
TROP-2	Trophoblast cell-surface antigen 2
TROP-2-ADC	TROP-2-directed antibody–drug conjugate
US	Ultrasound
WHO	World Health Organization
wtBRCA1/2	BRCA1/2 wild-type
