# Supplementary material for: Guidance for Canadian Breast Cancer Practice: National Consensus Recommendations for the Systemic Treatment of Patients with Triple Negative Breast Cancer in Both the Early and Metastatic Setting 2025
Source: Curr Oncol. 2026 Apr 24;33(5):243. doi: 10.3390/curroncol33050243 (PMC13205814; doi:10.3390/curroncol33050243)
Supplement: Supplementary file 1 [file curroncol-33-00243-s001.zip › curroncol-4149562-supplementary.pdf]

Table S1: Literature search strategy

	A	B	C	D
1	**Last search:** 2025-02-13<br>**Search terms:** (breast cancer) AND (TNBC) OR (triple negative)<br>**Source database:** Pubmed<br>**Filters:** English, RCT, 2010 and beyond<br>**Hits**: 475 articles<br>**Additional filters:** More than 50 pts<br>**Appended articles from Conferences:** ASCO (6), ESMO (14)<br>**Appended articles from guidelines**: ESMO (5), NCCN (69)<br>**Total:** 569 raw; 225 after filtering	**Neoadjuvant Studies**<br>ADC: 2<br>Boosters: 2<br>Everolimus: 2<br>ChT: 14<br>mAbs: 14<br>PARPi: 5<br>Platin: 11<br>Predictive markers: 8<br>PIK3CA: 1	**Adjuvant studies**<br>Capecitabine: 9<br>ChT: 27<br>mAbs: 9<br>Platin: 3<br>Predictive markers: 8	**Metastatic studies**<br>ADC: 8<br>Capecitabine: 1<br>CDK4/6i: 2<br>ChT: 52<br>mAbs: 25<br>New inh: 3<br>PARPi: 6<br>PIK3CA: 5<br>Platin: 6<br>TKI: 2

Table S2: Voting Results

National Consensus Recommendations for TNBC

Voting results (Agree with statement as is, Agree with statement with edits, Do not agree with statement, Abstain)

		Consensus Recommendation	Consensus was reached on:
1.	**Early Treatment – general approach**<br><br>For all aspects of care for patients with TNBC, HR-positive, or HER2-positive breast cancer, shared decision-making in partnership with patients and their caregivers is essential. All subsequent recommendations should be applied within the context of the patient's preferences, values, and goals, ensuring a collaborative approach to treatment planning. [Strong recommendation]	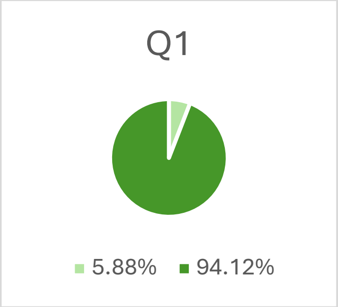<br>1 abstained<br>N=17	1st round
2.	Breast cancers that are ER ≤ 10%, PR ≤10%, and lack of HER2 overexpression or gene amplification (IHC ≤1+ or 2+ with fluorescence in situ hibridization (FISH) negative) should be treated as TNBC. [Moderate recommendation]	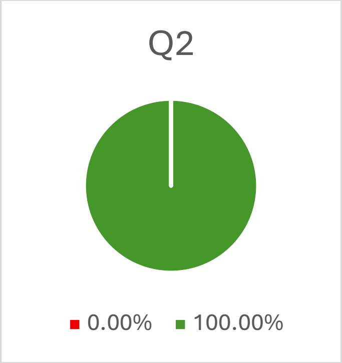	2nd round

		N=15	
3.	Patients with TNBC should be offered enrollment in clinical trials where appropriate, regardless of disease stage. [Expert opinion]	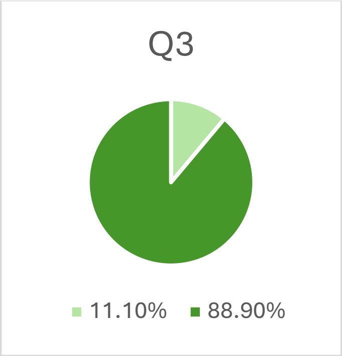 N=18	1st round
4.	Hereditary cancer gene panel testing should be offered to all patients with TNBC, regardless of age or family history. [Strong recommendation]	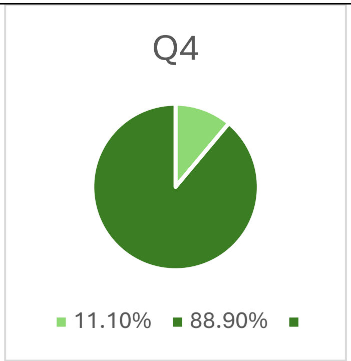 N=18	1st round

5.	Patients with early-stage TNBC should receive initial treatment (surgery or systemic neoadjuvant therapy) as quickly as possible, ideally within 30 days of biopsy. [Strong recommendation]	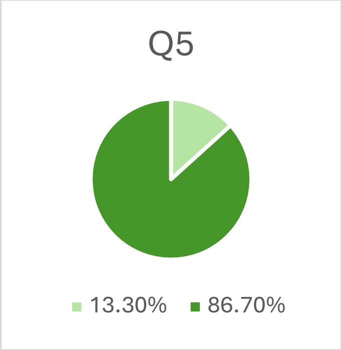<br>N=15	2nd round
6.	**Early treatment – Localized TNBC (tumours ≤2 cm (T1a-T1c) and N0 [Stage I]**<br><br>For patients with stage I TNBC whose tumour is ≤1 cm and N0 confirmed by ultrasound or ultrasound-guided FNA, upfront surgery is the standard of care. [Strong recommendation]	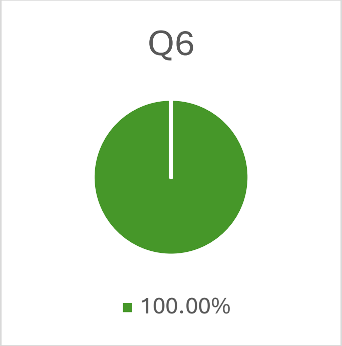<br>N=18	1st round
7.	For patients with stage I TNBC whose tumour is ≤0.5 cm (T1a) and whose disease is N0, adjuvant chemotherapy is not recommended. [Strong consideration]	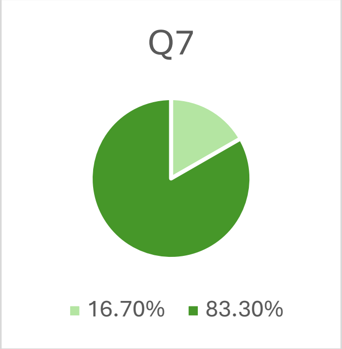<br>N=18	1st round

8.	For patients with stage I TNBC whose tumour is >0.5 cm and ≤1.0 cm (T1b), adjuvant chemotherapy should be considered through shared decision-making. [Strong or consideration]	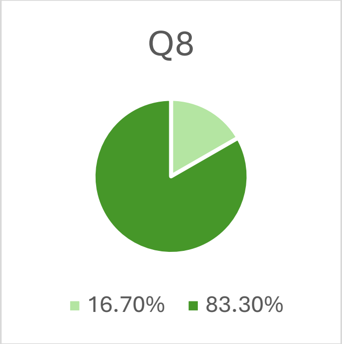<br>N=18	1st round
9. a)	For patients with stage I (T1a–T1c, N0) TNBC receiving adjuvant chemotherapy:<br><br>a. Standard therapy should include a taxane (e.g., paclitaxel or docetaxel). [Strong recommendation]	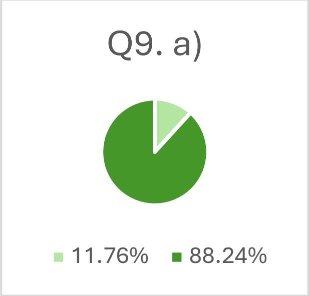<br>1 abstained<br>N=17	1st round
9. b)	For patients with stage I (T1a–T1c, N0) TNBC receiving adjuvant chemotherapy:<br><br>a. Anthracycline-containing regimens should generally be avoided, particularly for tumours ≤1 cm, to reduce the risk of long-term cardiotoxicity. For tumours >1.5 cm, anthracyclines may be considered. [Moderate recommendation]	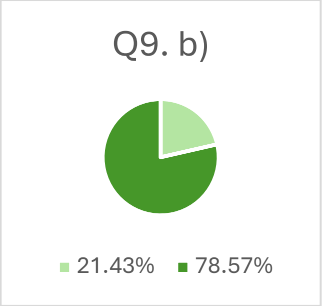<br>1 abstained<br>N=14	2nd round

10.	Although upfront surgery is preferred for those patients with stage I TNBC whose tumour is >1.0 cm and ≤2 cm (T1c) N0, neoadjuvant chemotherapy without immunotherapy may be considered. [Moderate recommendation]	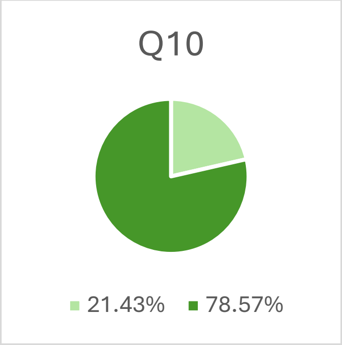<br>1 abstained<br>N=14	To be updated
11. a)	a) For patients who do not carry germline BRCA mutation who are treated with neoadjuvant chemotherapy (without immunotherapy), and who have residual disease identified at time of surgery, adjuvant capecitabine is recommended. [Strong recommendation]	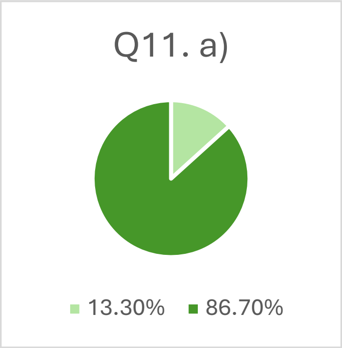<br>N=15	2nd round
11. b)	b) For patients who carry germline BRCA mutation who are treated with neoadjuvant chemotherapy (without immunotherapy), and who have residual disease at the time of surgery, adjuvant olaparib is recommended. (Strong recommendation)		2nd round

		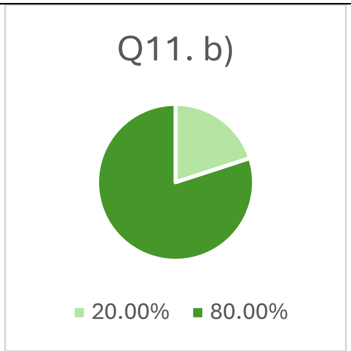<br>N=15	
12.	**Early treatment - TNBC (tumours >2 cm and/or any N [Stage II and III])**<br><br>In patients with Stage IIB-IIIC TNBC, initial staging includes imaging of chest/abdomen/pelvis and bone. [Strong recommendation]	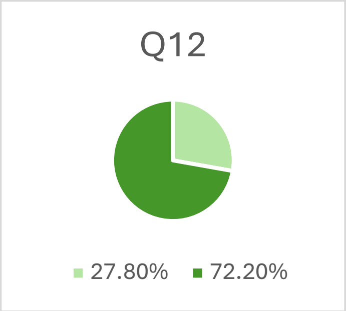<br>N=18	1st round
13.	For patients with clinical stage II or III TNBC, neoadjuvant chemotherapy with paclitaxel plus carboplatin followed by AC, with pembrolizumab administered throughout, is recommended. [Strong recommendation]	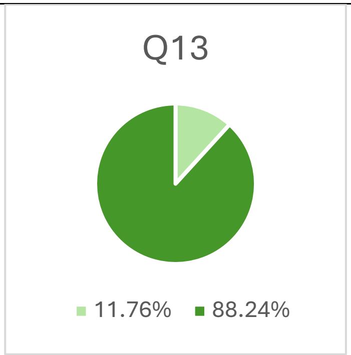<br>1 abstained<br>N=18	1st round

14.	For patients with stage II or III TNBC where a pathologic complete response (pCR) occurs following neoadjuvant pembrolizumab plus chemotherapy, continuation of adjuvant pembrolizumab alone to complete a total of one year of therapy is recommended, unless there is access to a de-escalation clinical trial. [Strong recommendation]	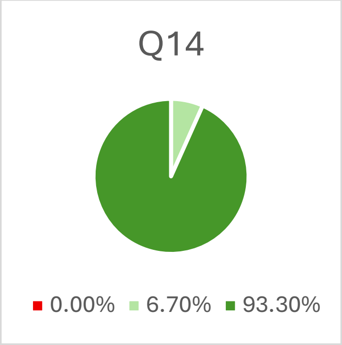<br>N=15	2nd round
15. a)	For patients with locally advanced or stage II/III TNBC who complete neoadjuvant pembrolizumab plus chemotherapy and have residual invasive disease on pathology, adjuvant systemic treatment should include:<br><br>a. Pembrolizumab plus a PARP inhibitor if gBRCA-mutated. [Strong consideration]	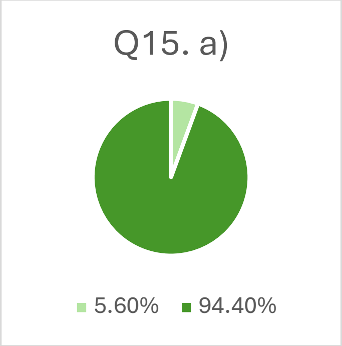<br>N=18	1st round
15. b)	For patients with locally advanced or stage II/III TNBC who complete neoadjuvant pembrolizumab plus chemotherapy and have residual invasive disease on pathology, adjuvant systemic treatment should include:<br><br>b. Pembrolizumab plus capecitabine if gBRCA wild-type. [Strong Consideration]	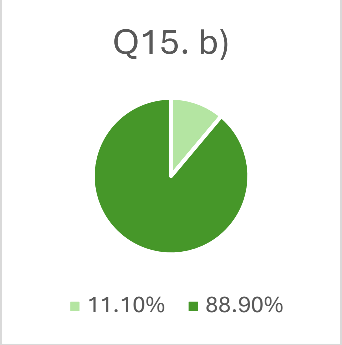<br>N=18	1st round

16.	For patients with stage II or III TNBC who have a contraindication to pembrolizumab, neoadjuvant chemotherapy—considering the inclusion of platinum agents—is recommended. [Strong recommendation]	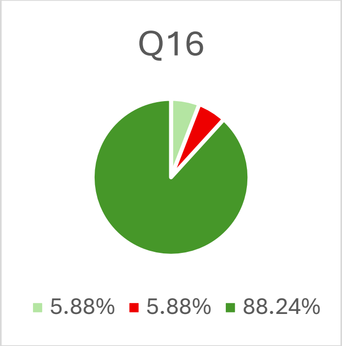<br>1 abstained<br>N=18	1st round
17.	For patients with higher-risk TNBC who did not receive neoadjuvant chemotherapy plus immunotherapy, adjuvant anthracycline- and taxane-based chemotherapy (e.g., dose-dense AC followed by paclitaxel) is recommended. [Strong recommendation]	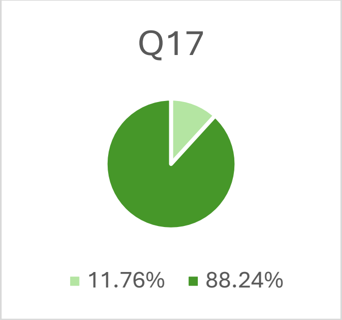<br>1 abstained<br>N=18	1st round
18.	In patients who receive neoadjuvant chemotherapy for TNBC and have a residual cancer burden (RCB) of 2 or 3, or a higher disease burden than initially assessed, repeat staging investigations after surgery should be considered. [Expert Opinion]	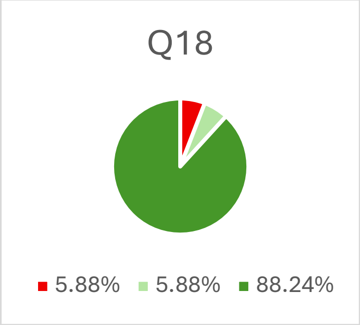<br>1 abstained	1st round

		N=18	
19. a)	For patients with TNBC who have completed adjuvant treatment:<br><br>a. History and physical examination should be performed every 3–6 months for the first 2 years, every 6 months in the third year, and annually thereafter. Annual mammography, with additional imaging as needed based on breast density, should also be performed. [Expert opinion]	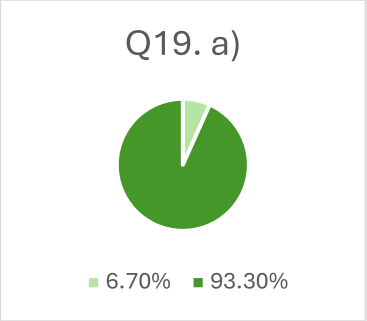<br>N=15	2nd round
19. b)	For patients with TNBC who have completed adjuvant treatment:<br><br>b. Follow-up assessments should include evaluation for signs and symptoms of recurrence or new ipsilateral/contralateral breast cancers, and monitoring for late effects of prior treatment (e.g., cardiac risk in those treated with anthracyclines, immune-related adverse events in those who received immunotherapy). [Expert opinion]	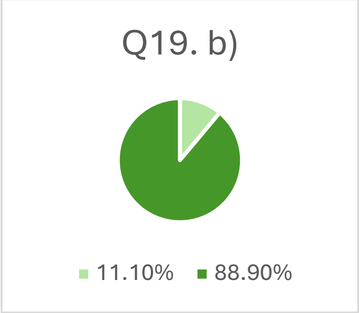<br>N=18	1st round

20.	**Early TNBC – special populations**<br><br>For patients with early-stage TNBC exhibiting uncommon histologic subtypes, management should be discussed in a multidisciplinary team (MDT) setting, including pathology review, to guide a tailored approach to surgery and systemic therapy. [Strong consideration]	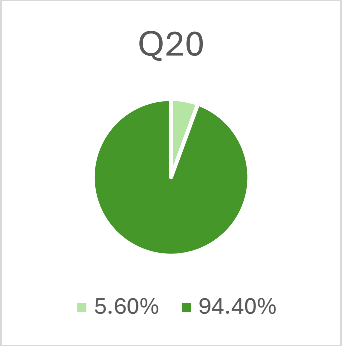<br>N=18	1st round
21.	For patients with ER-low (1-10%) HR+/HER2- breast cancer, adjuvant endocrine therapy +/- CDK4/6i should be discussed, though the absolute benefit is lower compared to more strongly ER+ tumours. Genomic testing may help guide decision-making in select cases. [Expert Opinion]	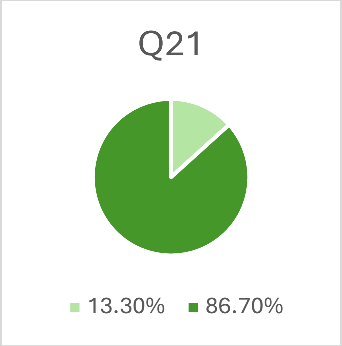<br>N=15	2nd round
22.	**Metastatic TNBC**<br><br>As consistently endorsed by the REAL Alliance across all disease sites (HER2+, HR+, TNBC), rebiopsy at first relapse is considered standard of care to confirm the diagnosis of recurrent breast cancer and reassess biomarker status (ER, PR, HER2). [Strong recommendation]	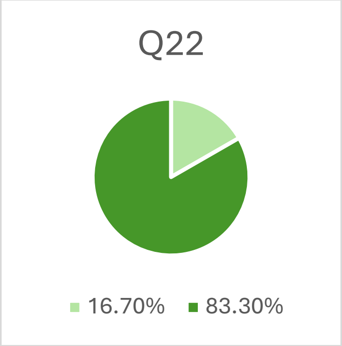<br>N=18	1st round

<table>
<tr><td>23. a)</td><td>For patients with a new diagnosis of metastatic TNBC:

a. Comprehensive staging with imaging of the chest, abdomen, pelvis, and bone, as well as brain imaging in patients with symptoms suggestive of central nervous system (CNS) involvement, is recommended. [Strong recommendation]</td><td>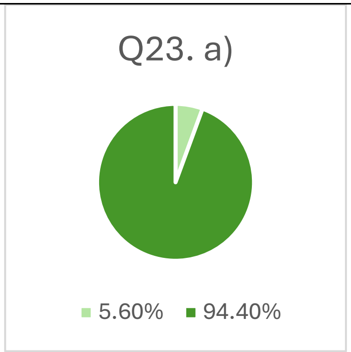

N=18</td><td>1st round</td></tr>
<tr><td>23. b)</td><td>For patients with a new diagnosis of metastatic TNBC:

b. Baseline brain imaging may also be considered in asymptomatic patients, with repeat assessment at the time of disease progression. [Expert opinion]</td><td>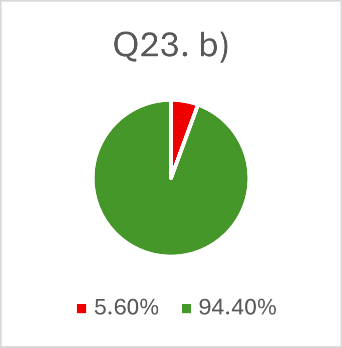

N=18</td><td>1st round</td></tr>
<tr><td>24.</td><td>Tumour PD-L1 status (CPS≥10) should be determined on the most informative tissue sample* for all patients with advanced/metastatic TNBC, if not previously tested or if prior negative result. [Strong recommendation]</td><td>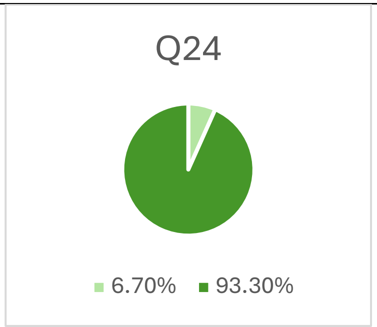

N=15</td><td>2nd round</td></tr>
</table>

25.	As consistently endorsed by the REAL Alliance across all disease sites (HER2+, HR+, TNBC), early discussion about goals of care and palliative support should be encouraged for patients with metastatic disease—particularly in cases of poor response to therapy or declining performance status—to ensure treatment aligns with patient values and optimizes quality of life. [Expert opinion]	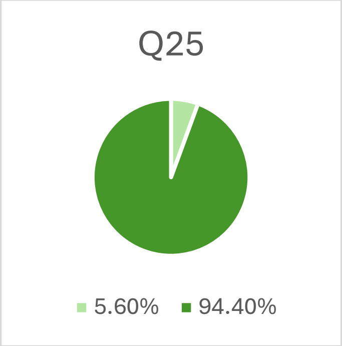<br>N=18	1st round
26. a)	For patients with metastatic TNBC in the first-line setting whose tumour is PD-L1 positive (CPS ≥10) and who have a disease-free interval of ≥1 year following adjuvant immunotherapy:<br><br>a. First-line pembrolizumab in combination with chemotherapy (paclitaxel, nab-paclitaxel, or carboplatin/gemcitabine) is currently recommended. [Strong recommendation]	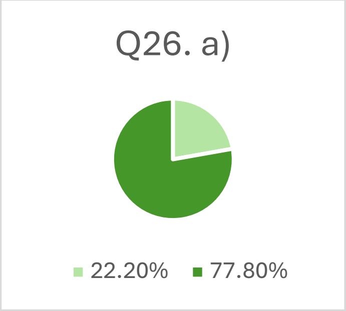<br>N=18	1st round
26. b)	For patients with metastatic TNBC in the first-line setting whose tumour is PD-L1 positive (CPS ≥10) and who have a disease-free interval of ≥1 year following adjuvant immunotherapy:<br><br>b. Pembrolizumab in combination with sacituzumab govitecan may also be considered in this setting, based on emerging data from ASCENT-04. [Strong consideration]	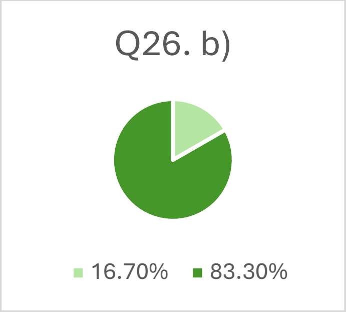<br>N=18	1st round

27. a)	For patients with locally recurrent or metastatic TNBC in the first-line setting whose tumors are PD-L1 negative (CPS <10) and who have relapsed within 6 months of prior adjuvant therapy:<br><br>a. First-line chemotherapy is recommended, with regimen choice guided by prior treatments, disease characteristics, disease-free interval, and patient preferences, incorporating shared decision-making. [Strong recommendation]	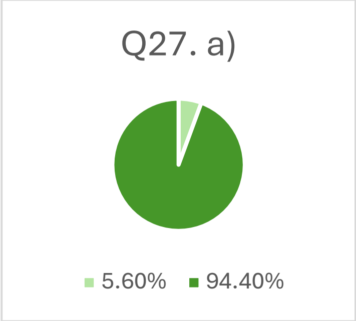 N=18	1st round
27. b)	For patients with locally recurrent or metastatic TNBC in the first-line setting whose tumors are PD-L1 negative (CPS <10) and who have relapsed within 6 months of prior adjuvant therapy:<br><br>b. Sacituzumab govitecan may also be considered in this setting, based on emerging data from the ASCENT-03 trial. [Conditional recommendation pending further validation]	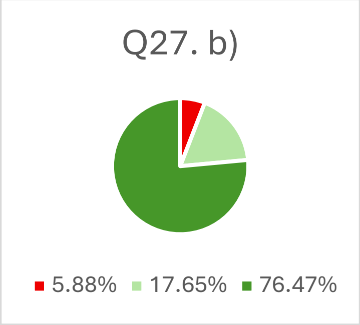 1 abstained<br>N=18	1st round
28.	For patients with metastatic TNBC in the first-line setting who have a germline BRCA mutation and a PD-L1–negative tumor (CPS <10), treatment with a PARP inhibitor is recommended. [Strong recommendation]	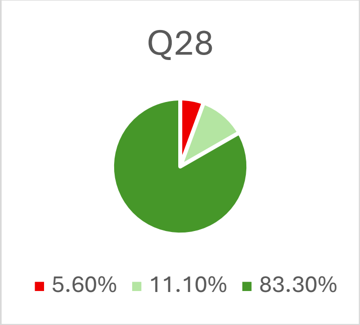 N=18	1st round

29.	For patients with metastatic TNBC whose disease has progressed on first-line chemotherapy (with or without immunotherapy), sacituzumab govitecan is the standard of care treatment. [Strong recommendation]	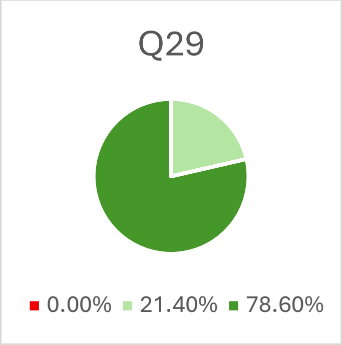<br>1 abstained<br>N=14	2nd round
30.	**3L and beyond**<br><br>For patients with metastatic TNBC whose disease has progressed on second-line treatment, selection of subsequent therapy should be based on prior treatments received in the metastatic setting. [Expert Opinion]	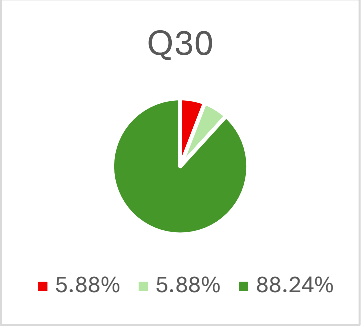<br>1 abstained<br>N=18	1st round
